# Drivers of methicillin-resistant *Staphylococcus aureus* (MRSA) lineage replacement in China

**DOI:** 10.1186/s13073-021-00992-x

**Published:** 2021-10-28

**Authors:** Hongbin Chen, Yuyao Yin, Lucy van Dorp, Liam P. Shaw, Hua Gao, Mislav Acman, Jizhen Yuan, Fengning Chen, Shijun Sun, Xiaojuan Wang, Shuguang Li, Yawei Zhang, Rhys A. Farrer, Hui Wang, Francois Balloux

**Affiliations:** 1 Department of Clinical Laboratory, Peking University People’s Hospital, Beijing, 100044 China; 2 UCL Genetics Institute, University College London, Gower Street, London, WC1E 6BT UK; 3 Department of Zoology, University of Oxford, Oxford, OX1 3SZ UK; 4 Medical Research Council Centre for Medical Mycology at the University of Exeter, University of Exeter, Geoffrey Pope Building, Stocker Road, Exeter, EX4 4QD UK; 5 The No. 971 Hospital of People’s Liberation Army Navy, Qingdao, 266000 Shandong China

**Keywords:** Methicillin-resistant *Staphylococcus aureus* (MRSA), Molecular evolution, Lineage replacement, Phylogeny, Phylogeography, Virulence

## Abstract

**Background:**

Methicillin-resistant *Staphylococcus aureus* (MRSA) is a major nosocomial pathogen subdivided into lineages termed sequence types (STs). Since the 1950s, successive waves of STs have appeared and replaced previously dominant lineages. One such event has been occurring in China since 2013, with community-associated (CA-MRSA) strains including ST59 largely replacing the previously dominant healthcare-associated (HA-MRSA) ST239. We previously showed that ST59 isolates tend to have a competitive advantage in growth experiments against ST239. However, the underlying genomic and phenotypic drivers of this replacement event are unclear.

**Methods:**

Here, we investigated the replacement of ST239 using whole-genome sequencing data from 204 ST239 and ST59 isolates collected in Chinese hospitals between 1994 and 2016. We reconstructed the evolutionary history of each ST and considered two non-mutually exclusive hypotheses for ST59 replacing ST239: antimicrobial resistance (AMR) profile and/or ability to colonise and persist in the environment through biofilm formation. We also investigated the differences in cytolytic activity, linked to higher virulence, between STs. We performed an association study using the presence and absence of accessory virulence genes.

**Results:**

ST59 isolates carried fewer AMR genes than ST239 and showed no evidence of evolving towards higher AMR. Biofilm production was marginally higher in ST59 overall, though this effect was not consistent across sub-lineages so is unlikely to be a sole driver of replacement. Consistent with previous observations of higher virulence in CA-MRSA STs, we observed that ST59 isolates exhibit significantly higher cytolytic activity than ST239 isolates, despite carrying on average fewer putative virulence genes. Our association study identified the chemotaxis inhibitory protein (*chp*) as a strong candidate for involvement in the increased virulence potential of ST59. We experimentally validated the role of *chp* in increasing the virulence potential of ST59 by creating Δ*chp* knockout mutants, confirming that ST59 can carry *chp* without a measurable impact on fitness.

**Conclusions:**

Our results suggest that the ongoing replacement of ST239 by ST59 in China is not associated to higher AMR carriage or biofilm production. However, the increase in ST59 prevalence is concerning since it is linked to a higher potential for virulence, aided by the carriage of the *chp* gene.

**Supplementary Information:**

The online version contains supplementary material available at 10.1186/s13073-021-00992-x.

## Background

Methicillin-resistant *Staphylococcus aureus* (MRSA) is ranked amongst the most serious multidrug-resistant threats [1] and are a major cause of nosocomial infections, ranging from superficial wound infections and food poisoning to pneumonia, infective endocarditis, bacteraemia and other systemic infections [2]. Methicillin resistance is conferred by the *mecA* gene, which is carried on a mobile genetic element (MGE) known as the SCC*mec* chromosomal cassette [3]. A divergent homologue of *mecA* first observed in 2011, *mecC*, can also confer methicillin resistance within a different SSC*mec* [4]*.* One prominent feature of MRSA is the strong population structure driven by the presence of multiple, essentially clonal, lineages of highly related strains. This population structure is typically described using sequence types (STs), defined as strains sharing the same sequence at a set of housekeeping genes used in multilocus sequence type (MLST) classification schemes [5].

Distinct MRSA lineages (STs) are found at markedly different prevalence globally. For example, ST239 is common in China [6–9], Singapore [10], Thailand [11] and other Asian countries [12–14], but rare in Europe and the Americas [15–17]. ST225 reaches a prevalence of between 60 and 80% of *S. aureus* in Central Europe but is virtually absent from the USA [18]. ST8 (USA300) is prevalent in the USA but is rarely observed in the rest of the world [16, 19]. ST22 is the most common lineage in the UK [15, 16, 20, 21] and has been identified at differing prevalence in parts of Asia [22–24].

Although some MRSA lineages remain at high prevalence in certain global regions for decades, the geographic distribution of many lineages is dynamic. Since the use of penicillin in the mid-1940s, multiple waves of new MRSA lineages have emerged and replaced previously established ones [25–28]. More recently, community-associated MRSA (CA-MRSA) have been replacing hospital-associated MRSA (HA-MRSA) as the dominant epidemic strains, with USA300 providing a prominent example [19, 29]. The mechanisms driving these phase transitions are poorly understood and remain best characterized for lineages frequently associated to nosocomial infections rather than commensal carriage in the wider population. Typical hospital-associated lineages include the ST239 and ST5 lineages, which carry the type II and III SCC*mec* elements, often lack the Panton–Valentine leukocidin (PVL) toxin genes, have relatively low virulence and usually infect people with poor immunity and/or during long-term hospitalization [30]. Conversely, community-associated lineages such as ST8, ST30 and ST59 carry the type IV and V SCC*mec* elements, often harbour the PVL toxin, and commonly infect young, otherwise healthy adults [19, 31–34].

Globally, ST239 isolates show signatures of convergent adaptation to hospital settings, including increased resistance to antimicrobials, increased ability to persist in healthcare environments and attenuated virulence [35, 36]. ST239 has historically been the dominant lineage in hospital infections in mainland China, where MRSA remains a particularly acute problem despite being in decline. Our multi-centre MRSA molecular surveillance schemes, together with work from Xiao et al. 2013 which considered different administrative regions, previously found that hospital-associated ST239 was the dominant MRSA lineage in China between 2005 and 2011, accounting for 50–80.8% of MRSA [6–8, 37], but has subsequently been declining in frequency (4.3%), with a general replacement of HA-MRSA STs by CA-MRSA STs [38]. CA-MRSA lineages, including ST59, have been increasing in prevalence since 2013 [24, 38–40], with recent work suggesting that between 2015 and 2017, ST59 has become the predominant MRSA clone (33%, 154/471) with ST59 accounting for more than half of CA-MRSA isolates [41]. The replacement of ST239 has been observed in other countries [42–44]. For example, Hsu et al. analysed the replacement of ST239 in Singapore by another HA-MRSA (ST22), likely after a single introduction in 2001, using phylogenetic reconstruction and comparative genomics [10]. However, the replacement of ST239 by CA-MRSA STs in China may have different drivers. In contrast to the situation in Singapore, the multiple STs involved in replacement events in China suggest the potential for more complex competitive interactions.

To understand the drivers of the ongoing replacement of MRSA clonal lineages in China, we have focused on the contrast between ST59 and ST239. We have previously shown that ST59-t437 has a fitness advantage over ST239-t030 in vitro [38], potentially contributing to this replacement. However, the same may not be true for other ST59 sub-lineages, for example, previously designated as the ‘Taiwan’ and ‘Asian-Pacific’ clones. Here, we investigate the comparative genomics of ST59 and ST239 in detail to better understand this replacement event and its potential consequences. We selected 132 ST239 and 72 ST59 *S. aureus* isolates from our surveillance network across 14 provinces for whole-genome sequencing (WGS). We reconstructed the evolutionary histories of these isolates through phylogenetic and accessory genome analyses. We additionally phenotyped all strains for their ability to produce biofilms and for their cytolytic activity, an indicator of virulence potential in a host. An association study using the presence and absence of virulence genes identified the chemotaxis inhibitory protein (*chp*) gene, a known contributor to human immune evasion in *S. aureus* [45], as amongst the strongest candidates for higher cytolytic activity in ST59. We experimentally validated the role of *chp* as a virulence factor in ST59 by creating Δ*chp* knockout mutants. Our results suggest that the ongoing MRSA lineage replacement in mainland China is linked to the higher virulence of ST59 strains relative to ST239, likely mediated through the carriage of the immune evasive *chp* gene, which is potentially of concern for future infections.

## Methods

### Bacterial isolates

We investigated the pathogen spectrum of bloodstream infections (BSI), hospital-acquired pneumonia (HAP) and intra-abdominal infections (IAI), prioritizing for this study ST59 and ST239 strains. We collected a total of 132 ST239 and 72 ST59 Chinese *S. aureus* isolates from 14 Chinese provinces between 1994 and 2016, sampled to cover a wide temporal and geographic range (Additional file 1: Table S1). The oldest 48 isolates were collected from a hospital in Beijing, because this was the only hospital participating in the surveillance effort that had stored bacterial strains prior to 2000. The remaining 156 isolates were collected from 30 hospitals across 14 provinces in China from 2002 to 2016.

**Fig. 1:**
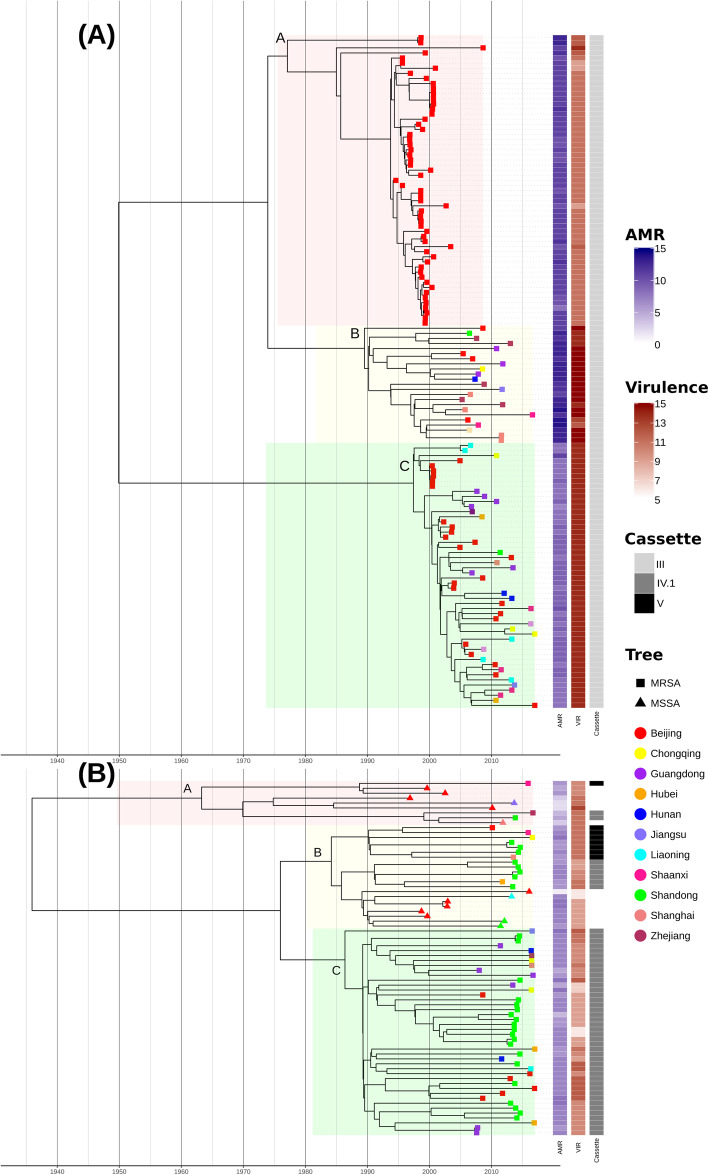
Maximum clade credibility (MCC) timed tree of Chinese ST239 (**A**) and ST59 (**B**) isolates. BEAST-inferred temporal phylogeny of Chinese *S. aureus* isolates. Tips are coloured according to the province of sampling. The highlighted boxes denote within ST sub-lineage structure for ST239 (ST239-A, ST239-B, ST239-C) and ST59 (ST59-A, ST59-B, ST59-C). The panel on the right provides the number (colour) of AMR and virulence (VIR) genes carried per isolate, together with the inferred cassette type for MRSA isolates

All of the 132 ST239 isolates belonged to MRSA and carried *mecA* located on a type III SCC*mec*. Amongst the 72 ST59 isolates, 14 (19.4%) were characterised as MSSA, and 58 (80.6%) were characterised as MRSA. Of the 58 ST59-MRSA isolates, eight (11.1%) carried the type V SCC*mec*, and the remaining 50 (88.9%) isolates carried the type IV SCC*mec* (Fig. 1).

In order to investigate the global geographic affinity of Chinese ST239 and ST59, we identified relevant genomes made available in published works as well as those released as Pathogenwatch projects associated to each ST type. In total, we included 153 global ST239 genome assemblies (downloaded from Pathogenwatch, https://pathogen.watch and published in previous studies [13, 44]) and 114 ST59 genomes. Of the 114 ST59 genomes, 13 were available via Pathogenwatch projects, and the remaining 101 genomes were downloaded from a previous study by Ward et al. [46].

### Whole-genome sequencing and multilocus sequence type assignment

Our 204 new *S. aureus* isolates were sequenced using 150-bp paired-end protocols on an Illumina NextSeq 500. All genomes were assembled de novo using SPAdes v3.10.0 [47]. The resultant contigs were inspected and then annotated using Prokka v1.12 [48]. MLST were assigned using PubMLST (https://pubmlst.org/saureus/).

### Preliminary phylogenetic analyses

The core genome was extracted from the annotated assemblies using Panaroo v1.1.2 [49]. Alignments were screened for recombination using ClonalFrameML [50], and the putative recombinant regions were removed before further phylogenetic analyses. Maximum likelihood phylogenetic trees were constructed in RAxML v8.2.10 using a GTR model and 1000 bootstrap replicates to assess the confidence at each node of the final tree [51] (Additional file 2: Fig. S1). The chromosomes of *S. aureus* isolates N315 (ST5) (GenBank accession no. NC_002745) and MW2 (ST1) (NC_003923) were used as outgroups to root the respective phylogenies as these represent the most closely related outgroups to Chinese and global ST239 and ST59. We tested for a temporal signal in the data (i.e. measurable evolution over the course of the sampling timespan) with a correlation between root-to-tip distances and the year of sampling in TempEst [52] and assessed significance following 10,000 date randomisations using the *roototip()* function implemented in BactDating [53] (Additional file 2: Fig. S2).

In parallel, we employed the same phylogenetic approach to construct a recombination pruned phylogenetic tree over the larger global datasets of ST239 and ST59 (Additional file 2: Fig. S3).

### Phylogeographic analyses

We used BEAST v2.4.7 [54] to estimate a timed phylogeny from the recombination-free core genome alignments of ST239 (3090 SNPs, 132 isolates) and ST59 (3423 SNPs, 72 isolates), including both tip dates and geographic information for each isolate. For both SNP alignments, the TN93 substitution model was selected based on the evaluation of all possible substitution models in bModelTest [55], and the number of invariant A, T, C and Gs sets to calibrate the clock rate estimation. All combinations of molecular clock models (strict, relaxed lognormal, relaxed exponential) and two population model priors (coalescent skyline, coalescent exponential) were evaluated. For each combination, three independent chains were run for 200,000,000 iterations sampling every 20,000 steps. Convergence of the Markov chain Monte Carlo (MCMC) chain was inspected in Tracer v1.6.0 and through evaluation of the effective sample sizes (ESS) and parameter value traces. Only runs with ESS > 200 were considered. The best-supported model following path sampling [56] was a skyline population model with a strict clock (Additional file 2: Table S2). The maximum clade credibility (MCC) tree under each model was generated in TreeAnnotator and plotted in ggtree [57].

### Accessory genome analyses of ST239 and ST59

The accessory genome was extracted using Panaroo v1.1.2 [49] (Additional file 2: Figs. S4-S5), and SCC*mec* types were determined as previously described [58]. Firstly, the specific SCC*mec* typing primer sequences were detected using BLAST, and then different combinations of cassette chromosome recombinases (*ccr*) gene complexes and the *mec* gene complex were analysed according to the definitions of the International Working Group on the Staphylococcal Cassette Chromosome Elements (IWG-SCC; www.sccmec.org). Prophage sequences within our assemblies were identified and characterized using the PHAge Search Tool web-server PHAST [59]. Contigs were defined as putative plasmids based on the presence of the *rep* gene or a previously identified plasmid sequence [60], and insertion sequences (IS) were identified and annotated using ISFinder [61]. Other MGEs, including mobile pathogenicity islands (SaPIs), genomic islands, mobile element structures (MES) and transposons, were determined using local ‘blastn’ searches, requiring a minimum query coverage value of 80% and a similarity threshold value of 95% (Additional file 2: Fig. S6, Additional file 2: Table S3). AMR and virulence genes were identified using ResFinder [62], VirulenceFinder [63] and the Virulence Factors DataBase [64] (VFDB), with a minimum query coverage value of 80% and a similarity threshold value of 90% (Fig. 1, Additional file 2: Figs. S7-S8). Resistance gene carriage and temporal patterns in the number of AMR genes carried was assessed using the Wilcoxon signed-rank test and linear regression (Additional file 2: Figs. S9-S12). The presence and phylogenetic association of the surface protein *sasX* in ST239 were also assessed (Additional file 2: Fig. S13).

### Methicillin resistance acquisition analyses

We employed nucleotide blast to identify the location of the SCC*mec* attachment site as defined in Noto et al. [65] in our ST59 Chinese *S. aureus* isolates (Additional file 2: Fig. S14). We extracted a maximal alignable region 500 bp upstream of, and including, the attachment site and aligned this across isolates using Clustalo v1.2.2 [66]. Hierarchical clustering was applied to the differences over this alignment using hclust in R [67].

### Biofilm formation test

**Fig. 2:**
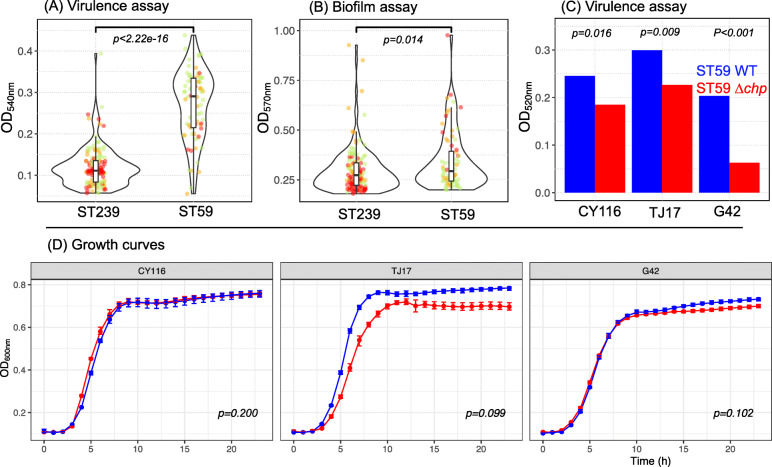
Phenotypic assays. **A** The overall virulence (OD_540_, *y*-axis) of 132 ST239 and 72 ST59 strains evaluated by lysis of erythrocytes by culture filtrates. Values for isolates of sub-lineage A, B and C are shown in red, orange and green respectively. **B** Biofilm formation ability (OD_570_; *y*-axis) of all 132 ST239 and 72 ST59 strains with sub-lineages coloured as in Fig. 1. **C** Cytolytic activity measured by lysis of erythrocytes by culture filtrates for wild-type (WT; blue) and Δ*chp* (*chp* knockout mutants; red) ST59 strains. Values provide the mean over three replicates per sample. **D** Growth curves for WT (blue) and Δ*chp* (red) ST59 strains. *p*-values are provided following the Wilcoxon signed-rank test (**A**–**C**) and following the permutation test (**D**), see the ‘Methods’ section

A total of 204 *S. aureus* strain was cultured overnight at 37 °C, and 0.5 McFarland bacterial suspension was prepared with sterile saline. Twenty microliters of the prepared bacterial suspension and 180 μl Tryptic Soy Broth (TSB) were added to each well of a 96-well microplate. Three parallel wells were prepared for each strain. TSB without bacteria suspension were used as a blank control. The 96-well microplate was placed at 37 °C for 24 h. The supernatant was gently discarded, and the 96-well plate was washed slowly twice with distilled water. The biofilm adhered to the microwells was fixed with 150 μl methanol for 20 min and then stained with 150 μl of 2% gentian violet stain for 15 min at room temperature. The 96-well plate was slowly washed twice with distilled water. Each well was decolorized by adding 150 μl of 95% ethanol for 30 min, and the absorbance at 570 nm of each well was measured using a spectrophotometer (Fig. 2, Additional file 1: Table S1).

### Lysis of erythrocytes by culture filtrates

A total of 204 *S. aureus* strains were cultured for 15 h in TSB. The bacterial culture was centrifuged at 5000 rpm for 5 min, and 10 μl of the supernatant was added into a 96-well plate containing 90 μl of PBS buffer per well. Then, 100 μl of human red blood cells (2% v/v in Dulbecco’s phosphate-buffered saline) was added to the supernatant and incubated at 37 °C for 1 h with gentle shaking. The culture was centrifuged at 1500 rpm for 12 min at 4 °C, without disturbing the cells. One hundred microlitres of the supernatant was transferred to a new 96-well plate, and the absorbance at 540 nm of the supernatant was measured using a spectrophotometer. This procedure was repeated twice to assess the concordance (Fig. 2, Additional file 1: Table S1).

### Pan-GWAS for ST59 virulence

To identify genes putatively associated with the potential for virulence in ST59, we fitted generalized linear models (GLMs) to the presence and absence matrix of virulence-associated genes (identified by VFDB) against the measurements obtained from the lysis of erythrocyte assay. Detected associations were reported for all genes passing a significance threshold of < 0.05 following Bonferroni correction (Additional file 2: Fig. S15, Additional file 2: Table S4).

### *chp* inactivation

*S. aureus chp* gene inactivation mutants were obtained for ST59 isolates spanning each sub-lineage: CY116, TJ17 and G41, using the pnCasSA-BEC genome editing system according to methods described previously [68]. Briefly, annealed *chp* spacer oligos were first inserted into the *Bsa*I sites of the pnCasSA-BEC plasmid by Golden Gate assembly. Then, the *chp* spacer-introduced pnCasSA-BEC plasmid was transformed into the wild-type *S. aureus* RN4220 strain using electroporation (2.1 kV mm^−1^, 100 Ω, 25 μF, 2-mm cuvette). The colonies were selected on a TSB agar plate containing 5 mg/L chloramphenicol at 30 °C. The *chp* spacer-introduced pnCasSA-BEC plasmid isolated from the RN4220 strain was transformed into target *S. aureus* CY116, TJ17 and G42 strains by electroporation (2.1 kV mm^−1^, 100 Ω, 25 μF, 2-mm cuvette). The colonies were also selected on a TSB agar plate containing 5 mg/L chloramphenicol at 30 °C. The successful *chp* inactivation strain was confirmed by PCR and sequencing. After confirmation, the *chp* spacer-introduced pnCasSA-BEC plasmid was cured by culturing the cells at 42 °C for 12 h without adding chloramphenicol.

### Growth assay

*S. aureus* wild-type and *chp* inactivated strains were cultured in TSB to achieve an OD_600_ of 0.6. The culture was diluted to OD_600_ = 0.01 and then cultured at 37 °C with agitation at 200 rpm for 24 h. The OD_600_ was measured every 30 min to obtain a growth curve. Growth curves were compared using a permutation test implemented by the *compareGrowthCurves()* function within statmod v1.434 (https://CRAN.R-project.org/package=statmod), specifying 50,000 permutations (Fig. 2, Additional file 2: Fig. S16).

### In vitro competition test

In vitro competition tests were performed as described previously [38]. Three pairs of ST59 wild strains (CY116, G42, TJ17) and *chp* knockout mutants (CY116-Δ*chp*, G42-Δ*chp*, TJ17-Δ*chp*), together with two randomly selected ST239 strains (W3200, 09B38), were diluted to 0.5 × 10^7^ colony-forming units (CFU)/ml. Paired, diluted ST59 and ST239 suspensions were mixed 1:1 in equal volumes. Then, 10 μl of the mixture was added to 20 ml of LB broth and cultured at 37 °C with shaking at 200 rpm. The 10-μl subcultures were transferred to fresh LB broth every 24 h. At the same time, 10-μl subcultures were inoculated on drug-free and 1 μg/ml rifampicin MH agar plates. The colonies of ST59 (susceptible to rifampicin) and ST239 (resistant to rifampicin) were counted after incubation overnight. After 96 h, the adaptive difference (*S*), relative adaptive fitness (*F*) and fitness cost (*C*) were calculated according to the following formulas (Additional file 2: Table S5).

$$ S=\ln \left[{\left(\frac{\frac{s_t}{r_t}}{\frac{s_{t-1}}{r_{t-1}}}\right)}^{\frac{1}{17}}\right] $$

Note: *s*_*t*_ is the number of sensitive colonies, and *r*_*t*_ is the number of resistant colonies.

$$ F=1+S $$

$$ C=\left(1-F\right)\times 100\% $$

## Results

### ST239 and ST59 have acquired extensive diversity since their emergence

Our sequenced *S. aureus* isolates comprise 132 ST239 and 72 ST59 collected between 1994 and 2016 from hospitals spanning 14 Chinese provinces (Additional file 1: Table S1). Phylogenetic analysis of the recombination-free core genome alignments of the ST239 and ST59 isolates identified within-ST diversity over 2313 and 2244 core genes, respectively. ST59 could be resolved into three phylogenetic sub-lineages which we term for reference ST59-A, ST59-B and ST59-C (Fig. 1). Comparing these to more traditional *spa* typing points to a rough agreement between sub-lineages and dominant *spa* type (ST59-A: t163, ST59-B: t437, ST59-C: t437; see Additional file 2: Fig. S1). Similarly, ST239 had three major sub-lineages (dominant *spa* types ST239-A: t037, ST239-B: t037, ST239-C: t030; see Additional file 2: Fig. S1), though with some within sub-lineage diversity in *spa* type assignment. The phylogenetic sub-lineage structure could not be fully explained by geographic sampling in either ST, with isolates collected from hospitals located in different Chinese provinces interspersed in the phylogeny (Fig. 1). The exception was the oldest sub-lineage of ST239 (ST239-A/ST239-t037), which comprised a set of isolates collected from the same hospital in Beijing between 1994 and 2000. Across the remaining isolates, the lack of a strong phylogenetic structure based on the location of the hospital suggests frequent acquisition and transmission events between sampled provinces.

Using the date of isolate collection to calibrate the respective phylogenies, we obtained a strong correlation between the time of sampling and the root to tip distances which were highly significant following 10,000 date randomisations (ST239: *R*^2^ = 0.84; ST59: *R*^2^ = 0.50; Additional file 2: Fig. S2). This allowed us to infer genome-wide substitution rates for the non-recombining portion of the ST239 and ST59 alignments of 1.8 × 10^−6^ (1.7 × 10^−6^–2.0 × 10^−6^, 95% HPD) and 1.2 × 10^−6^ (1.0 × 10^−6^–1.4 × 10^−6^) substitutions per nucleotide site per year, respectively (see the ‘Methods’, Additional file 2: Table S2). While these estimates indicate a slightly faster rate in ST239, both fall largely in line with previous evolutionary rates reported for *S. aureus* which range between 1.2 × 10^−6^ and 4.0 × 10^−6^ substitutions per nucleotide site per year [13, 18, 21, 28, 35, 44]. Bayesian inference of a timed phylogeny across isolates [54] suggests that both ST groups have been in joint circulation from the early to mid-twentieth century, prior to the clinical introduction of methicillin. The sampled Chinese ST239 sub-lineages were estimated to last share a common ancestor in approximately 1950 (ST239: 95% HPD 1943–1956) (Fig. 1A), and the three sampled sub-lineages of 72 ST59 isolates were estimated to have diverged from each other in the mid-1930s (ST59: 1936, 95% HPD 1923–1948; Fig. 1B).

We expanded both datasets to include isolates from worldwide publicly available collections [13, 44, 46] and released as projects on Pathogenwatch (Additional file 2: Fig. S3, see the ‘Methods’ section). Within these global phylogenies, we again observed considerable substructure, highlighting that within-ST diversity is not a unique property of our sampled collection of strains from China but a more general feature of the ST59 and ST239 *S. aureus* lineages. Across the global core genome phylogenies, we observed that our considered Chinese isolates recapitulated a proportion of the diversity observed globally (Additional file 2: Fig. S3A, Additional file 2: Fig. S3B) consistent with multiple introductions from and into China, likely with distinct phylogeographic origins.

### Accessory genome structure

The accessory genome represents an important component of the total *S. aureus* genome, harbouring elements involved in mediating virulence, immune evasion and resistance, which can be acquired by horizontal gene transfer. The accessory genome is not necessarily conserved within a particular ST and includes genes such as SCC*mec*, prophages, *S. aureus* mobile pathogenicity islands (SaPIs), insertion sequence (IS) elements, transposons and plasmids [69]. Having characterized the core genome of our Chinese *S. aureus* isolates, we compared the accessory genomes of each ST in more depth.

Our ST239 and ST59 isolates had large and variable accessory genomes of 3206 and 3026 genes, respectively (Additional file 2: Fig. S4). For ST239, the shared accessory genome was clustered into three groups, corresponding to the sub-lineages identified by the core genome phylogeny (Additional file 2: Fig. S5A). Variation in the accessory genome (presence/absence) suggests changes took place in the accessory genome of lineages of ST239 during its more recent evolution (Additional file 2: Fig. S5A). Though all ST239 isolates in our dataset carried the SCC*mec* of type III together with a Tn*5801* like a transposon and an IS*256* (Fig. 1A, Additional file 2: Fig. S6A, Additional file 2: Table S3). The presence of MGEs in the ST59 Chinese isolates was less variable across sub-lineages than for ST239 and did not recapitulate the core genome structure (Additional file 2: Fig. S5B, Additional file 2: Fig. S6B). The majority (*n* = 50) of ST59 isolates carried SCC*mec* of type IV.1, while a further eight carried SCC*mec* of type V and 14 had no SCC*mec* (Fig. 1B).

### Evolutionary dynamics of antimicrobial resistance in ST239 and ST59

Given that isolates were sampled from hospitals across China, a possible hypothesis for the different trajectories observed between STs could be susceptibility to antimicrobial treatment. For instance, one a priori hypothesis would be that the decline in the prevalence of ST239 in China [6, 7, 31] may derive from a lesser ability to survive antimicrobial treatment. The latter for instance has been shown to have contributed to the success of different clonal complexes, for instance, the replacement of CC30 by CC22 following the emergence of fluoroquinolone resistance [21]. We considered the presence, absence and total carriage of AMR genes in both of our ST239 and ST59 cohorts over the 22-year surveillance period (Fig. 1, Additional file 2: Fig. S7, Additional file 2: Figs. S9-10). We previously showed that a subset of ST239 isolates was more phenotypically resistant than ST59 isolates [38]. This finding was recapitulated from comparative genomics: ST239 isolates instead carried significantly more AMR elements on average than the ST59 isolates (mean: 10.3 vs. 6.3), a trend which held when considering only MRSA isolates (Fig. 1, Additional file 2: Fig. S9).

There was however considerable heterogeneity in the carriage of genes conferring resistance to macrolides (*erm(A*)) and aminoglycosides (*ant(6)-Ia* and *aph(3’)-III*) within ST239 (Additional file 2: Fig. S7A), though based on current sampling, we detected an overall trend of decreasing resistance element carriage over time largely driven by elevated AMR carriage in ST239-A (adj. *R*^2^ = 0.05; *p* = 0.008) (Fig. 1A, Additional file 2: Fig. S10a). Conversely, we found no change in the number of AMR genes carried by ST59 over the sampling period (Additional file 2: Fig. S10a).

All our ST239 isolates were methicillin-resistant. However, the ST59 dataset included a small subset of methicillin-susceptible *S. aureus* (MSSA) isolates (Fig. 1B, Additional file 2: Fig. S6B, Additional file 2: Fig. S14A). MSSA and MRSA (cassette types IV.1 and V) isolates were interspersed across the core genome phylogeny, consistent with ST59-MRSA evolving through repeat acquisitions of the SCC*mec* element. Alternatively, some ST59-MRSA strains may have secondarily lost the SCC*mec* element. To formally test this, we extracted and aligned a maximal region extending 500 bp upstream of the SCC*mec* attachment site [65]. The diversity in this flanking region formed three clear clusters which matched the cassette type assignments (Additional file 2: Fig. S14B-C). The most parsimonious scenario for this clustering requires at least five independent acquisitions of the SCC*mec* given our dataset of 58 MRSA ST59. We also infer one likely instance of secondary gene loss (Additional file 2: Fig. S14A), due to the lack of SCC*mec* in a single isolate clustered within a well-supported (100% bootstrap support) MRSA phylogenetic clade.

### Virulence and biofilm production of ST59 and ST239 strains

In silico prediction of virulence potential is more challenging than for AMR profiles [70], with counts of putative virulence factors likely a poor proxy for phenotypic virulence. The number of known virulence factors detected in ST239 was highly structured by core genome sub-lineage (Fig. 1A, Additional file 2: Fig. S8). Given our sampling, ST239 isolates harboured on average significantly more putative virulence genes than ST59 (mean 12.8 vs. 10.0; Wilcoxon signed-rank test *p* < 0.0001; Additional file 2: Fig. S11), and the total number of virulence genes carried across ST239 significantly increased over sampling time (adj. *R*^2^ = 0.62; *p* < 0.0001), with ST239-A carrying significantly fewer virulence factors than each of ST239-B or ST239-C (Additional file 2: Figs. S11-S12). We observed no such temporal trend in ST59 (Additional file 2: Fig. S12B). Within ST59, only a subset of isolates carried the PVL cytotoxin encoded by *lukF-PV* and *lukS-PV* (Additional file 2: Fig. S8B). The *S. aureus* surface protein SasX has also been shown to enhance nasal colonization and virulence in skin and lung infection models [71]. All isolates in both lineage ST239-A and ST239-B carried *sasX*, while only one strain of lineage ST239-C (the more recent lineage of ST239) carried *sasX* (Additional file 2: Fig. S13).

We next assessed the potential for virulence in the host using lysis of erythrocyte assay and the ability to form biofilms via a Tryptic Soy Broth (TSB) assay (see the ‘Methods’ section). Phenotypes were recorded for all the 132 ST239 and 72 ST59 strains that we sequenced (Additional file 1: Table S1). Despite carrying on average fewer virulence-associated genes compared to ST239, ST59 strains had significantly higher cytolytic activity in vitro than their ST239 counterparts (Kruskal-Wallis test, *p* < 0.0001; Fig. 2A). Conversely, the ability to produce biofilms was only slightly increased in ST59 (Fig. 2B).

### Identification and functional validation of *chp* as a driver of virulence

To identify candidate genes associated to the increased cytolytic activity in the ST59 lineage, we performed an association study using the presence/absence of virulence genes and results from the lysis of erythrocyte assay. We identified, amongst the top five genes most strongly associated to the erythrocyte lysis phenotype, members of the immune evasion complex and, in particular, the chemotaxis inhibitory protein (*chp*) gene (Additional file 2: Fig. S15, Additional file 2: Table S4). The *chp* gene encodes the chemotaxis inhibitory protein of staphylococci (CHIPS), a protein which blocks neutrophil chemotaxis and contributes to the evasion of the innate immune response [72]. CHIPS has been identified as a candidate for virulence in ST59 strains [73–75], and the ability to evade immune defence, through the carriage of *chp*, offers a possible explanation for the increased relative fitness of a lineage. Hence, we performed functional analyses to validate the statistical association between the carriage of the *chp* gene and the phenotypic measurements in our ST59 dataset.

We selected three ST59 strains, representing the diversity of sub-lineages (annotated ST59-A, ST59-B, ST59-C), to construct *chp* knockout mutants, and compared the virulence between wild-type strains and *chp* knockout mutants. Cytolytic activity of all three strains decreased significantly after *chp* knockout (Fig. 2C), supporting its role in enhancing the virulence potential of ST59 compared to ST239. Knockout of the *chp* gene led to no significant change in the growth rates of the mutants, though we observe a small, though not significant (*p* = 0.10), decrease in the growth rate of TJ17-Δ*chp* (Fig. 2D). We next measured the relative fitness of the three knockout Δ*chp* strains and their wild-type progenitors in competition assays against two *S. aureus* ST239 strains and observed no statistically significant differences in colony counts between wild-type and knock-out strains following 96 h of growth (Additional file 2: Fig. S16, Additional file 2: Tables S5). These results suggest that carriage of *chp* has no detectable fitness cost to ST59.

## Discussion

MRSAs remain a major nosocomial problem [76], in particular, in China [77]. Globally, the distribution of MRSA STs is highly structured and characterized by serial emergence and replacement events of new strains [30]. Until recently, HA-MRSA lineages such as ST239 have dominated in Asia [78], likely aided by their ability to evade treatment by methicillin [79]. However, in China, ST239 MRSAs have been in marked decline over the past two decades, while CA-MSSA and CA-MRSA lineages including ST59 are becoming more prevalent [31, 38]. In vitro characterisations have indicated a lower competitive ability of ST239 relative to CA-MRSA lineages ST30, ST8 and ST59 [35, 38], though somewhat counter-intuitively, with ST239 less susceptible to antibiotic treatment than replacing lineages such as ST59 [38]. As a result, the specific genotypic and phenotypic drivers underlying the displacement of ST239 by CA-MRSA lineages in China, and what the consequences of these may be for pathogenicity, remain poorly understood.

In this work, we generated paired genomic and phenotypic data for ST239 and CA-MRSA ST59 isolated from patients in Chinese hospitals spanning 22 years, covering the time course of lineage replacement. We estimated the age of emergence of both STs to the early to mid-twentieth century, suggesting both evolved prior to the first clinical use of methicillin before going on to acquire extensive pan-genome diversity (Fig. 1). Consistent with prior phenotypic observations, we found that the declining ST239 carried significantly more resistance elements than ST59, reinforcing that susceptibility to treatment is not an important driver of changing patterns of CA-MRSA prevalence in this case. This mirrors the situation in Singapore where a less drug-resistant lineage, ST22, is increasing in prevalence [44]. Consistently, we also detected no trend of increasing genotypic AMR carriage in ST59 over the roughly 20-year sampling period.

We therefore tested two hypotheses to explain the dynamics of ST replacement in China: increased transmission through longer persistence on surfaces due to biofilm production or higher virulence, for instance, through the ability to evade early innate immune defences. Despite the identification of large and highly structured accessory genomes, we found no evidence for biofilm production driving lineage replacement (Fig. 2). Conversely, we observed that ST59 strains had markedly enhanced cytolytic activity in vitro compared to ST239, consistent with the higher virulence of CA-MRSA strains more broadly (Fig. 2). We identified the *chp* gene as a strong contributor to the enhanced virulence potential of ST59 in our dataset. In support of the mechanistic role of *chp* in ST59, we found that the presence of *chp*, when compared to *chp* knockouts, increased the expression of other *S. aureus* virulence factors, including other members of the innate immune evasion cluster (*sea*, *sak*, *scn*).

We suggest that enhanced virulence is likely to play an important role in the success of ST59 in China. However, we note that virulence is a complex and context-dependent trait. For example, a simple sum of the number of putative virulence elements is unlikely to be a good proxy for the total infective potential of a CA-MRSA or HA-MRSA *S. aureus*. Consistently, we observed no relationship between the decrease in the prevalence of ST239 in Chinese hospitals and the number of predicted virulence elements over the sampling period. In fact, as with the number of resistance elements, ST239 carried on average a larger number of putative virulence factors than ST59, with the number of virulence determinants tending to increase over time, while remaining constant in ST59 over the same period. Our results therefore do not suggest a mere count of elements previously associated with clinically relevant phenotypes can explain differing patterns of ST incidence in China.

We do however highlight the importance of taking the variable accessory genome into account for the identification of virulence-associated factors and AMR determinants. For instance, although we saw no overall trend towards increasing resistance gene carriage (count), we inferred that ST59 independently acquired methicillin resistance at least five times in the 1980s (Additional file 2: Fig. S14). While the acquisition of methicillin resistance has been detected prior to the therapeutic use of methicillin [31] in the 1960s, our estimates of the times of SCC*mec* acquisition by MSSA ST59 is more consistent with a scenario where horizontal transfer was driven by the selective pressure of clinical drug use. This is also consistent with reports of high mobility of SCC*mec* between MSSA and MRSA strains in a recent hospital outbreak [80].

Virulence as a measurable trait ultimately requires evaluation using in vivo models though cytolytic activity, which can be readily measured using lysis of erythrocyte assays, offers a good proxy given hemolysins are well-established virulence factors [81]. Higher cytolytic activity as a virulence mechanism in our ST59 isolates is consistent with pronounced measurements of virulence in ST59 lineages [82] and the role of the innate immune evasion cluster in mouse infection models [83]. In our dataset, we identified, through a knock-out experiment, that the mechanism enhancing the virulence of ST59 in China can be well explained by the carriage of a single gene, encoding CHIPS (*chp*). *chp* is located on the β-hemolysin (hlb)-converting bacteriophages, which drives instant immune evasion by *S. aureus* [72]. While fitness costs have been associated to the acquisition of resistance genes [27, 35, 84–86], including large SCC*mec* elements, we detect no such effect with *chp* when comparing the growth rates of wild-type to knockout ST59 strains.

Enhanced virulence activity has recently been suggested as a contributor to the increasing prevalence of another CA-MRSA ST5 in China, including in MSSA [83]. Our findings lend further support to this hypothesis, with the carriage of *chp* as a potentially major contributor to the ongoing prevalence of ST59 strains in China, likely at the expense of ST239. This is concerning as CHIPS ability to evade the early immune response through both inhibitions of chemotaxis and phagocytosis [72], suggests a potential for more severe and longer-term infections. However, it is probable that other factors also contribute. For instance, it is important to remember that surveillance data informing the replacement of MRSA lineages is primarily based on severe infections in hospitals caused by different MRSA lineages. MRSAs are also found at fairly high prevalence in the nares of healthy carriers [87], possibly at different frequencies than in nosocomial infections. As such, the high relative virulence of the ST59 lineage may more directly reflect its incidence in nosocomial MRSA infection, rather than its prevalence in the wider population. In addition, there may be some stochasticity in which STs rise in prevalence when previously dominant STs decline. One possibility is that documented loss of fitness of ST239 [35] has also played an important role in the dynamics of which CA-MRSA are now being observed.

Our work highlights the full potential of WGS in microbial genomics when analysed in conjunction with associated phenotypes. Far too often, genomic sequence data is deposited on publicly available databases without the associated metadata, even when available. This greatly limits the potential of genomic sequence data reuse to address further questions. We hope that this work will contribute to encouraging others to make their phenotypic data fully accessible. In principle, similar, larger studies could be conducted on publicly available data to address pressing questions on the genetic basis of other key phenotypic traits in major human pathogens.

## Conclusions

By analysing the core and accessory genomes of a carefully phenotyped set of isolates collected in China over the last two decades, we were able to identify the likely driver of the replacement of HA-MRSA lineages by their CA-MRSA counterparts in China. We found no evidence for a role of AMR or the ability to colonise and persist in the environment. Conversely, we found that the expanding ST59 lineage was far more virulent than its previously widespread nosocomial ST239 counterpart. We could further show that this difference in virulence is likely largely driven by the carriage of the *chp* gene by ST59, an important contributor to immune evasion. We surmise that the ongoing lineage replacement observed in Chinese hospitals is primarily driven by differences in virulence of different MRSA lineages, which can in part be explained by the carriage of the *chp* gene by the ST59 lineage.

## Supplementary Information

**Additional file 1: Table S1.** 132 ST239 and 72 ST59 Chinese isolates included in the phylogenetic analysis. MLST, multilocus sequence type.

**Additional file 2: Fig. S1.** Core genome phylogeny for ST239 and ST59. **Fig. S2.** Regression of root-to-tip distance against sampling time for core genome alignments. **Fig. S3.** Global core genome phylogenies for ST239 and ST59. **Fig. S4.** Shared core and accessory genes amongst Chinese ST239 and ST59 isolates. **Fig. S5.** Conservation of accessory genome homology groups (HGs) across the Chinese ST239 and ST59 isolates. **Fig. S6.** Presence/absence of mobile genetic elements (MGEs) amongst Chinese ST239 and ST59 isolates. **Fig. S7.** Presence/absence of antimicrobial resistance genes amongst Chinese ST239 and ST59 isolates. **Fig. S8.** Presence/absence of virulence genes amongst Chinese ST239 and ST59 isolates. **Fig. S9.** Number of resistance (AMR) genes carried between ST sub-lineages. **Fig. S10.** Changing trends of antimicrobial resistance (AMR) mutations/elements within ST239 and ST59 lineages over time. **Fig. S11.** Number of virulence genes carried between ST sub-lineages and within ST239 and ST59 sub-lineages. **Fig. S12.** Trends of virulence mutations/elements within Chinese ST239 and ST59 lineages over time. **Fig. S13.** Core genome phylogeny for ST239 annotated for *sasX*. **Fig. S14.** Methicillin resistance acquisition analyses. **Fig. S15.** Presence absence of virulence genes against cell lysis phenotype. **Fig. S16.** Colony counts at 0 hours and 96 hours for three ST59 isolates (wild-type and knock-out) compared to two possible ST239 control strains. **Table S2.** Inferred tMRCA, clock rates and marginal likelihoods inferred by Bayesian dating analyses. **Table S3.** The distribution of mobile genetic elements (MGEs) in different sub-lineages. **Table S4.** Bonferroni corrected *p*-values following fitting of a generalized linear model (GLM) for VFDB virulence gene presence / absence against cell lysis phenotype. **Table S5.** Relative adaptive difference (S), fitness (F) and fitness cost (C) estimated from the *in vitro* competition experiment between ST59-WT and ST59 Δ*chp*.

## Data Availability

Raw Illumina short-read data from the 204 newly generated *S. aureus* isolates are available on NCBI with BioProject ID: PRJNA491594 (https://www.ncbi.nlm.nih.gov/bioproject/PRJNA491594) [88]. Full accompanying metadata and phenotypic information are available in Additional file 1: Table S1.
