# Supplementary material for: Drivers of methicillin-resistant Staphylococcus aureus (MRSA) lineage replacement in China
Source: Genome Med. 2021 Oct 28;13:171. doi: 10.1186/s13073-021-00992-x (PMC8555231; doi:10.1186/s13073-021-00992-x)
Supplement: Supplementary file 2 — Additional file 2: Fig. S1. Core genome phylogeny for ST239 and ST59. Fig. S2. Regression of root-to-tip distance against sampling time for core genome alignments. Fig. S3. Global core genome phylogenies for ST239 and ST59. Fig. S4. Shared core and accessory genes amongst Chinese ST239 and ST59 isolates. Fig. S5. Conservation of accessory genome homology groups (HGs) across the Chinese ST239 and ST59 isolates. Fig. S6. Presence/absence of mobile genetic elements (MGEs) amongst Chinese ST239 and ST59 isolates. Fig. S7. Presence/absence of antimicrobial resistance genes amongst Chinese ST239 and ST59 isolates. Fig. S8. Presence/absence of virulence genes amongst Chinese ST239 and ST59 isolates. Fig. S9. Number of resistance (AMR) genes carried between ST sub-lineages. Fig. S10. Changing trends of antimicrobial resistance (AMR) mutations/elements within ST239 and ST59 lineages over time. Fig. S11. Number of virulence genes carried between ST sub-lineages and within ST239 and ST59 sub-lineages. Fig. S12. Trends of virulence mutations/elements within Chinese ST239 and ST59 lineages over time. Fig. S13. Core genome phylogeny for ST239 annotated for sasX. Fig. S14. Methicillin resistance acquisition analyses. Fig. S15. Presence absence of virulence genes against cell lysis phenotype. Fig. S16. Colony counts at 0 hours and 96 hours for three ST59 isolates (wild-type and knock-out) compared to two possible ST239 control strains. Table S2. Inferred tMRCA, clock rates and marginal likelihoods inferred by Bayesian dating analyses. Table S3. The distribution of mobile genetic elements (MGEs) in different sub-lineages. Table S4. Bonferroni corrected p-values following fitting of a generalized linear model (GLM) for VFDB virulence gene presence / absence against cell lysis phenotype. Table S5. Relative adaptive difference (S), fitness (F) and fitness cost (C) estimated from the in vitro competition experiment between ST59-WT and ST59 Δchp. [file 13073_2021_992_MOESM2_ESM.pdf]

Supplementary Materials: Additional File 2

Drivers of methicillin resistant *Staphylococcus aureus* (MRSA) lineage replacement in China

Hongbin Chen[1,2,a], Yuyao Yin[1,a], Lucy van Dorp[2,a*], Liam P. Shaw[2,3], Hua Gao[1], Mislav Acman[2], Jizhen Yuan[1,5], Fengning Chen[1], Shijun Sun[1], Xiaojuan Wang[1], Shuguang Li[1], Yawei Zhang[1], Rhys A Farrer[2,4], Hui Wang[1*], Francois Balloux[2*]

[1] Department of Clinical Laboratory, Peking University People's Hospital, Beijing, 100044, China.

[2] UCL Genetics Institute, University College London, Gower Street, London, WC1E 6BT, UK.

[3] Nuffield Department of Medicine, John Radcliffe Hospital, University of Oxford, Oxford, OX3 9DU, UK.

[4] Medical Research Council Centre for Medical Mycology at the University of Exeter, University of Exeter, Geoffrey Pope Building, Stocker Road, Exeter, EX4 4QD, UK.

[5] The no.971 Hospital of People's Liberation Army Navy, Qingdao, Shandong, 266000.

[a] These authors contributed equally to the work.

*Corresponding authors.

Supplementary Figures

(A)

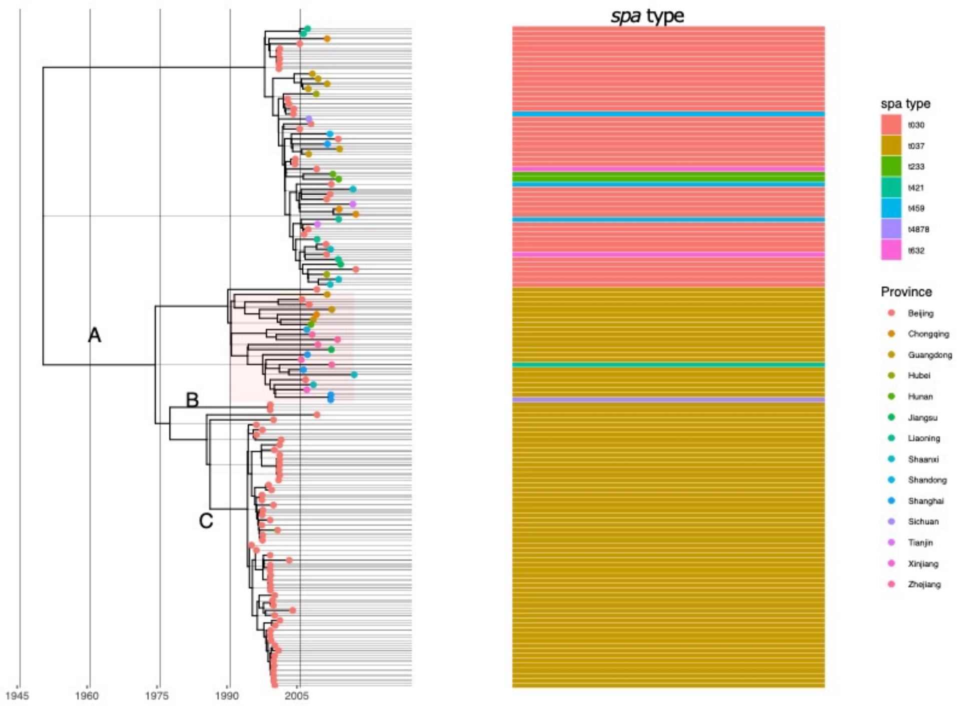

(B)

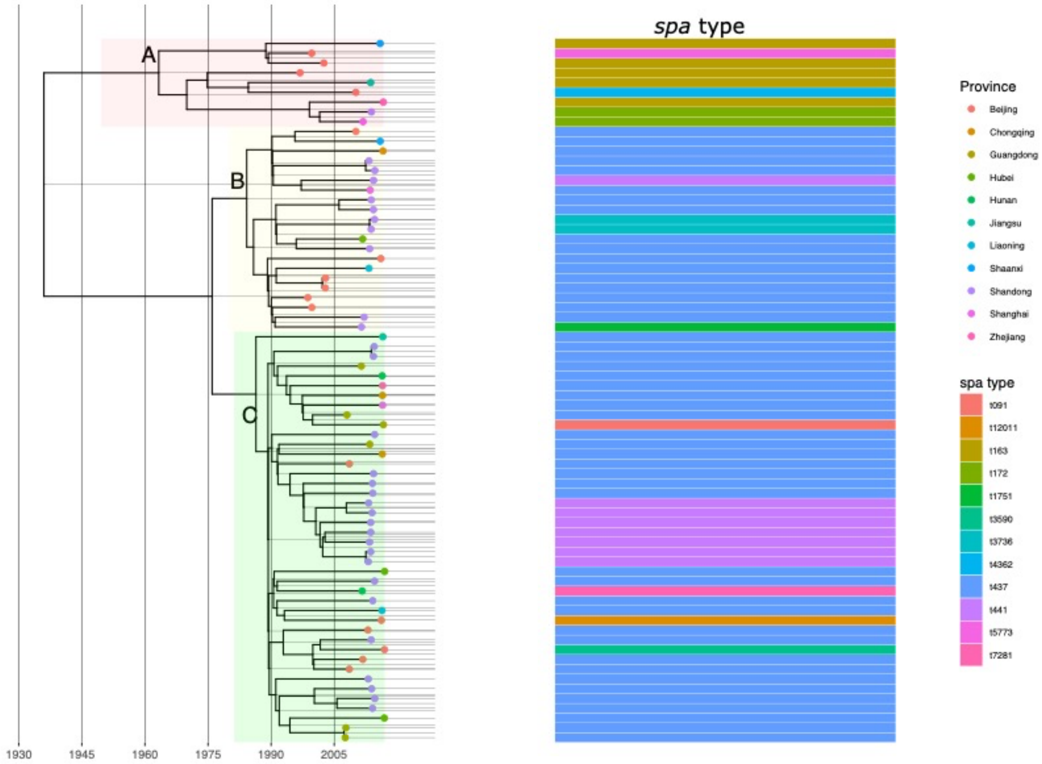

Fig S1: Core genome phylogeny for ST239 (A) and ST59 (B) with the coloured panel at right providing the concordance with sub-typing schemes, as per the coloured legend.

a) ST239 Rate=2.34e−02,MRCA=1949.89,R2=0.84,p<1.00e−04

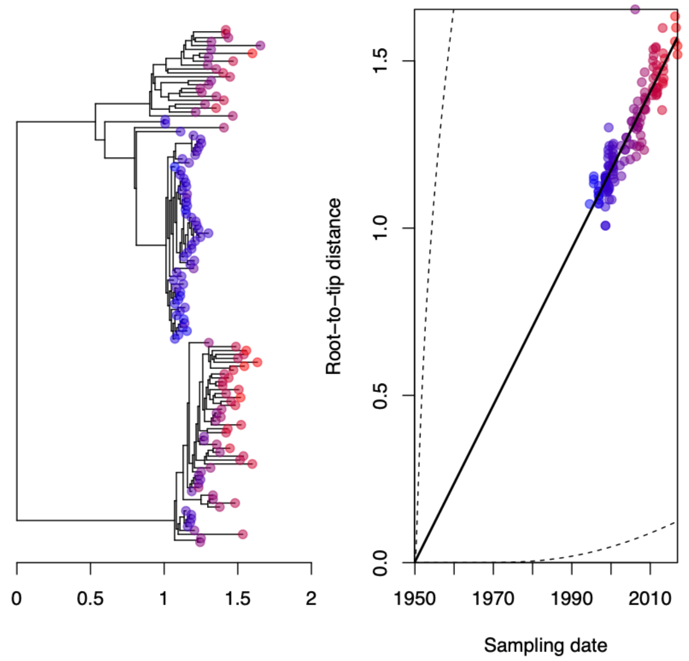

b) ST59 Rate=4.27e−03,MRCA=1939.57,R2=0.49,p<1.00e−04

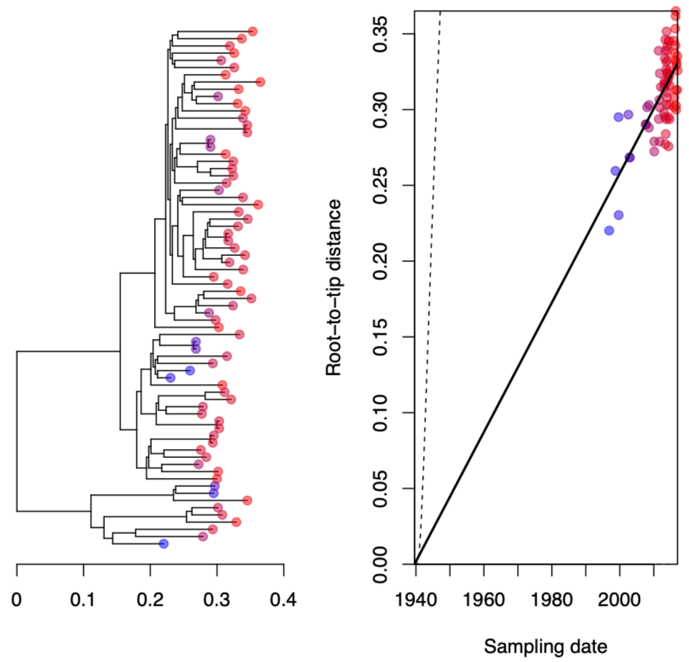

Fig S2: Regression of root-to-tip distance against sampling time for core genome alignments for Chinese (A) ST239 and (B) ST59 isolates. P-values provide the significance following 10,000 date randomisations.

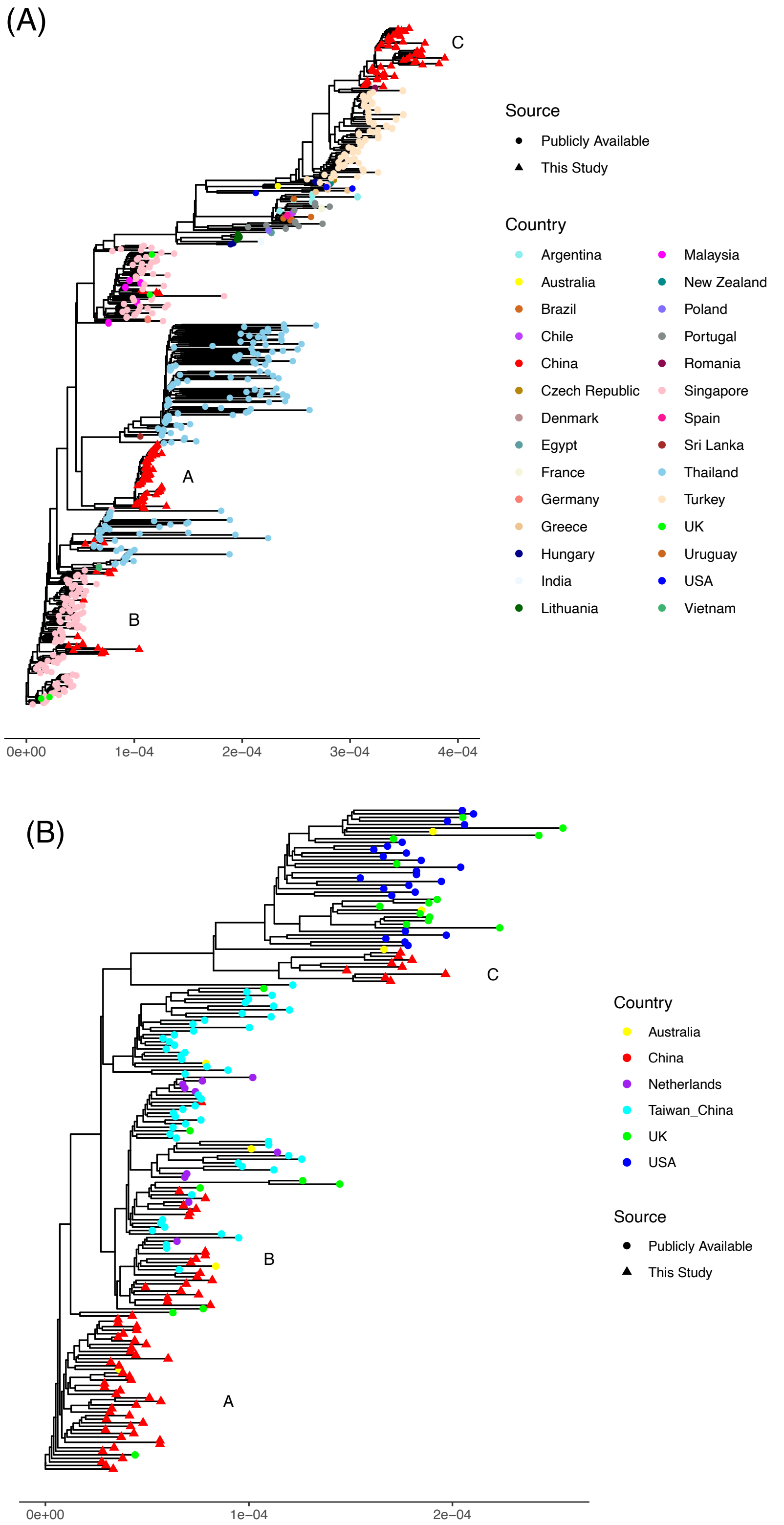

Fig S3: Global core genome phylogenies for (A) ST239 and (B) ST59. Unrooted global core genome phylogenies for (A) ST239, based on 1564 core genes (n=285, sampled over 28 countries) and (B) ST59, based on 2158 core genes (n=186, sampled over 6 countries). The phylogenetic placement of Chinese isolates newly generated in this study (triangle symbol) is indicated.

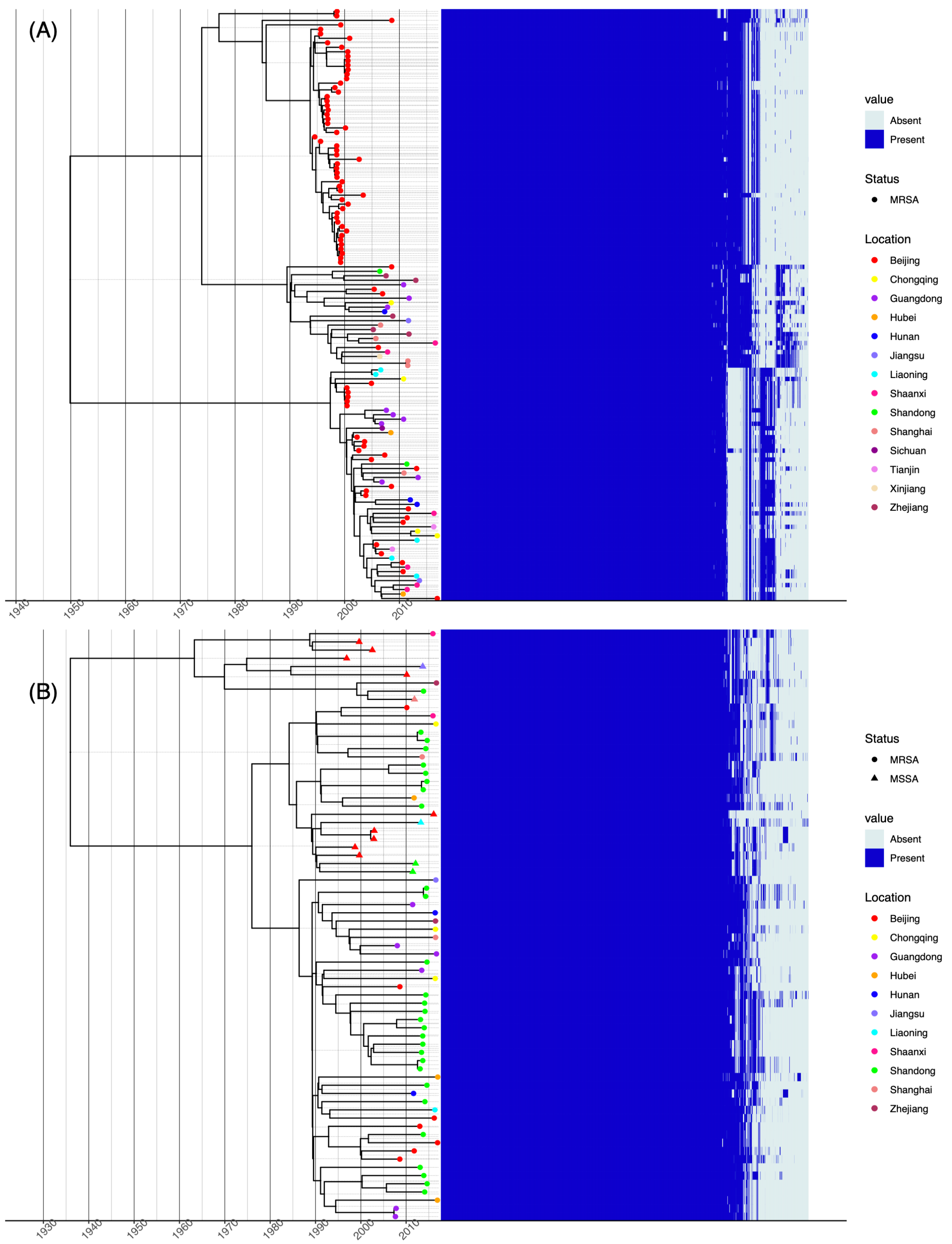

Fig S4: Shared core and accessory genes amongst Chinese (A) ST239 and (B) ST59 isolates. Core and accessory genes were identified using Panaroo v1.1.2. The heatmap provides the presence and absence (see legend) of genes ordered as per the core phylogeny. ST239 comprised 2404/3206 core to total genes. ST59 comprised 2317/3026 core to total genes.

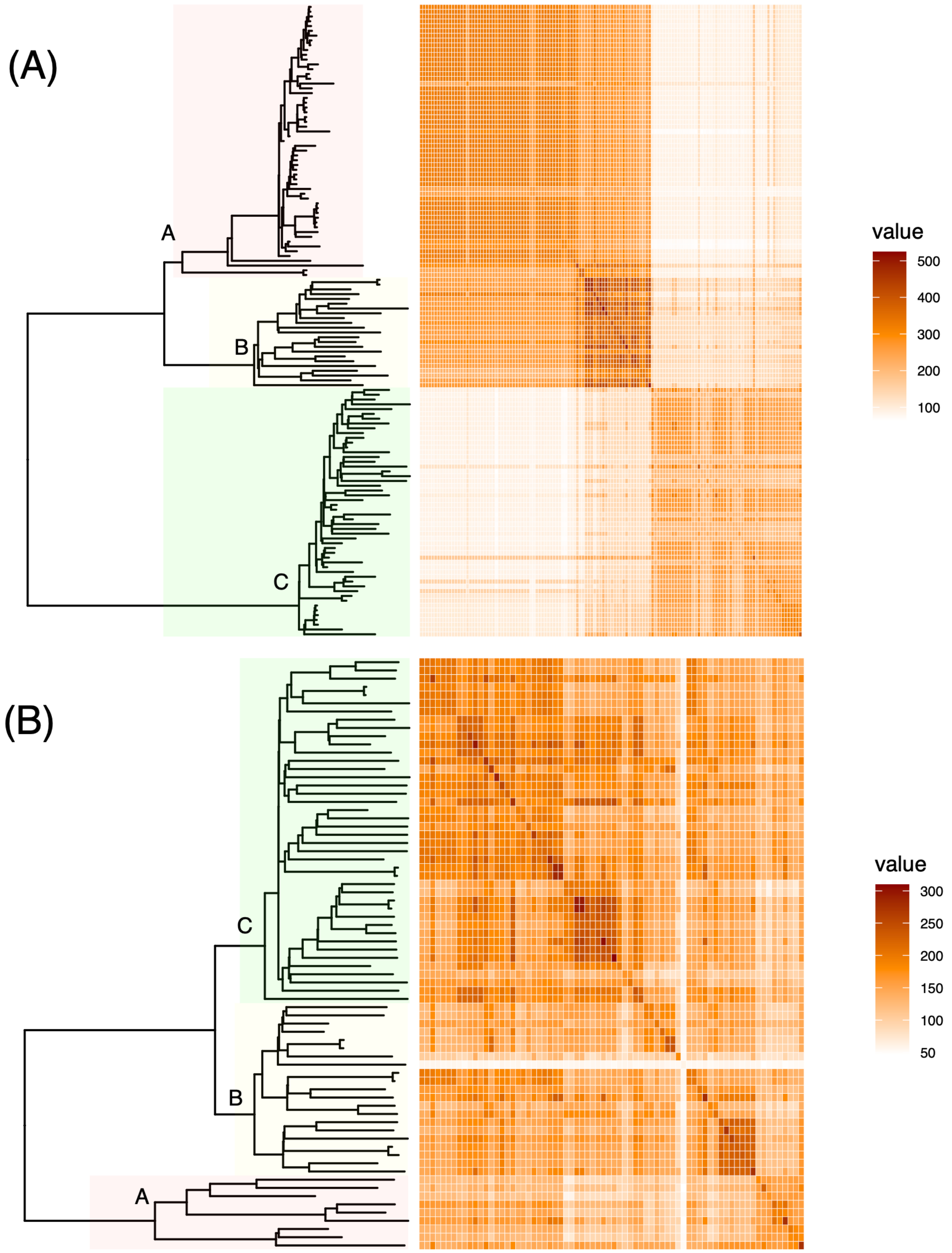

Fig S5: Conservation of accessory genome homology groups (HGs) across the Chinese (A) ST239 and (B) ST59 isolates. Isolates were ordered according to the phylogeny shown on the left of the figure. The conserved HGs in the pairwise comparison are shown by the heatmap, coloured according to the number of conserved HGs per pair (scale). One ST59 isolate (CY10) only carried 61 accessory genes despite a mean accessory component across all other ST59 of 230 (170-300 95% CI) accessory genes.

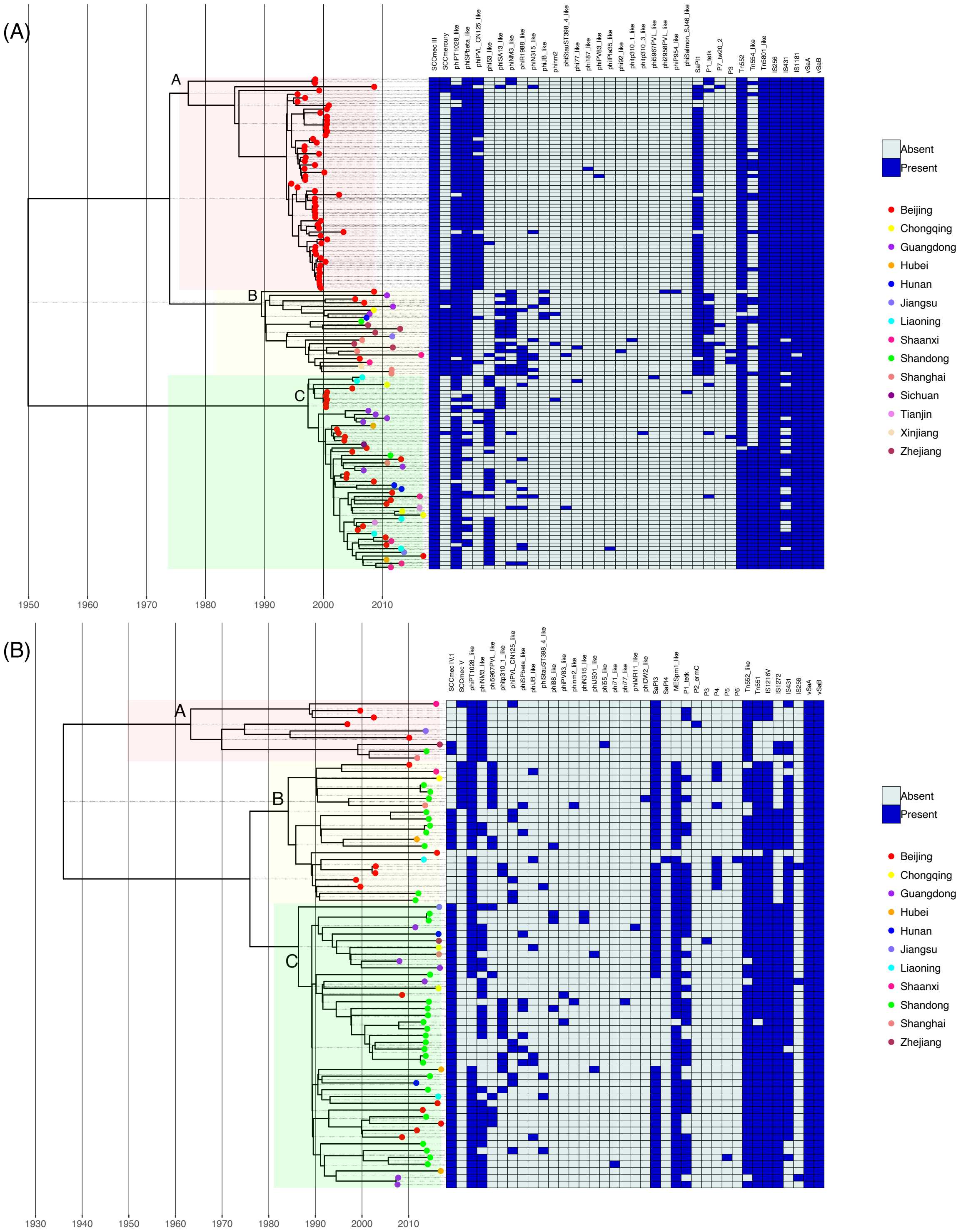

Fig S6: Presence/absence of mobile genetic elements (MGEs) amongst Chinese (A) ST239 and (B) ST59 isolates. Maximum clade credibility timed trees of (A) ST239 and (B) ST59 populations based on BEAST analysis. The tips of the tree are coloured according to the province where isolates were sampled. The presence of an MGE in an isolate is denoted by the heatmap, as given in the legend. Included MGEs are: SCC*mec*, SCC*mercury*, prophages, plasmids, Staphylococcal Pathogenicity Islands (SaPI), mobile element structures (MES), transposons, insertion sequences (IS) and genomic islands.

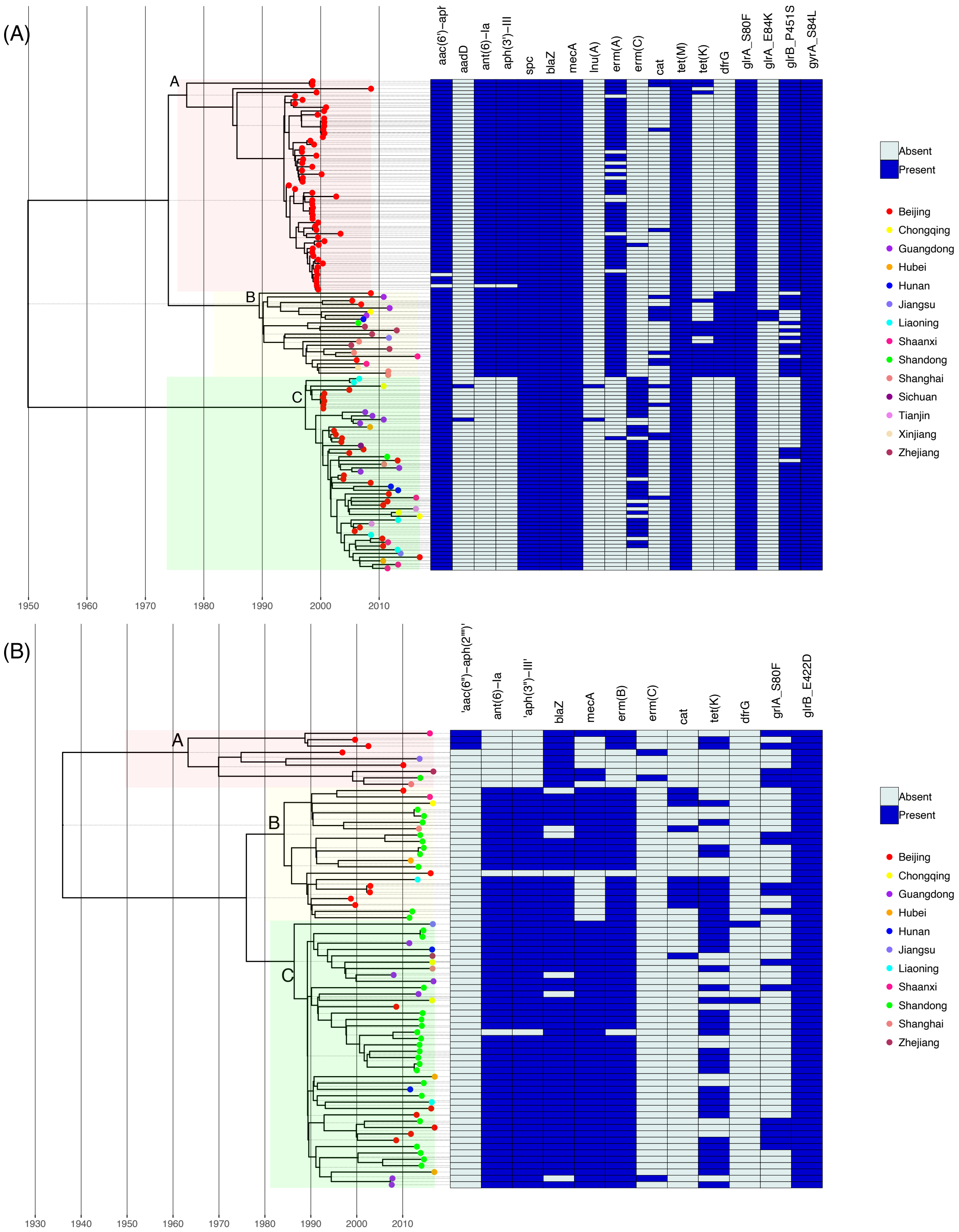

Fig S7: Presence/absence of antimicrobial resistance genes amongst Chinese (A) ST239 (A) and (B) ST59 isolates. Maximum clade credibility timed trees of (A) ST239 and (B) ST59 populations based on BEAST analysis. The tips of the tree are coloured according to the province where isolates were sampled. The presence of an AMR gene is denoted by the heatmap and given in the legend. Included genes occur at least once per cohort.

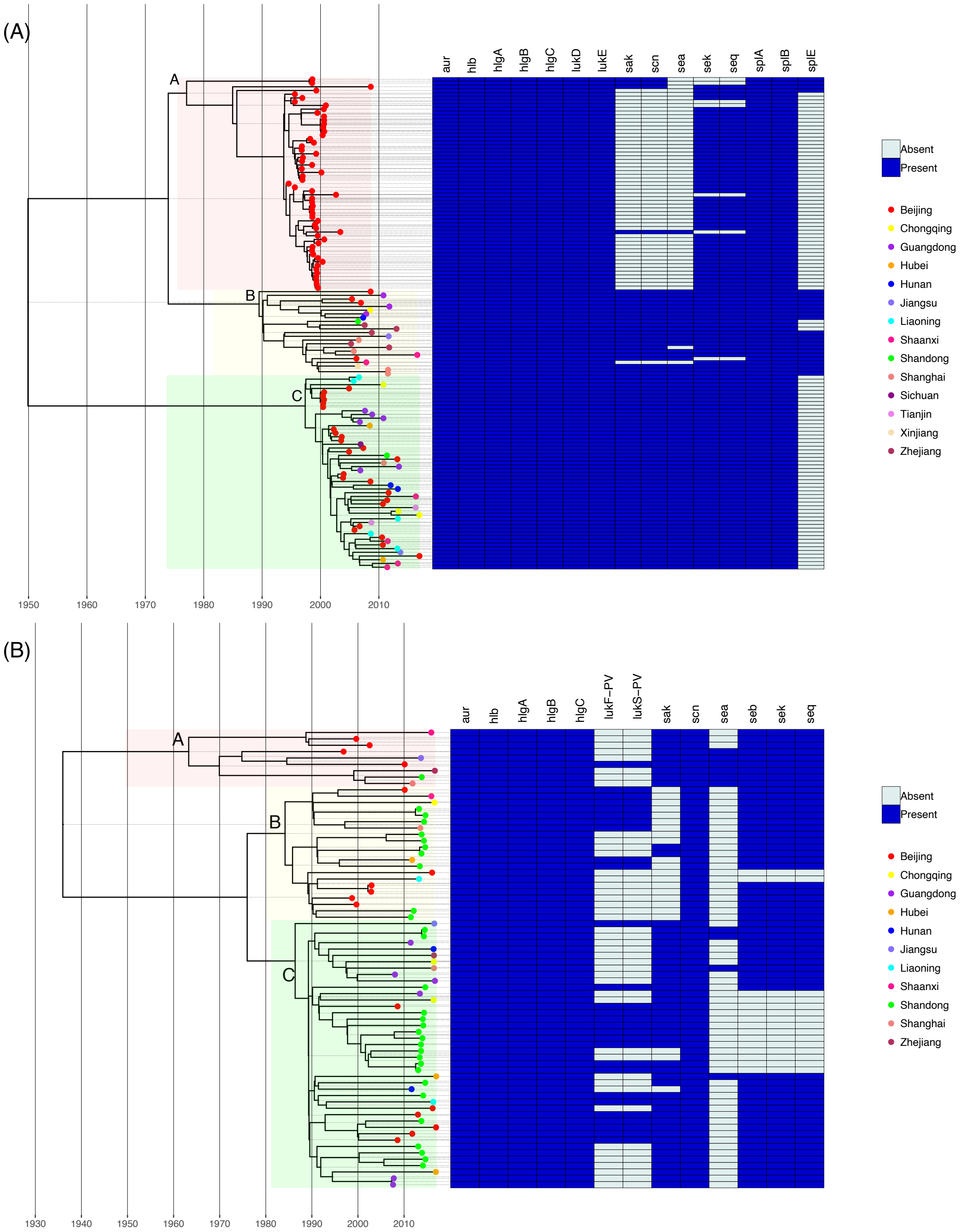

Fig S8: Presence/absence of virulence genes amongst Chinese (A) ST239 and (B) ST59 isolates. Maximum clade credibility timed trees of (A) ST239 and (B) ST59 populations based on BEAST analysis. The tips of the tree are coloured according to the province where isolates were sampled. The presence of a virulence gene, that occurs at least once per cohort, is denoted by the heatmap and given in the legend. Virulence genes were annotated against the VirulenceFinder database (VFDB).

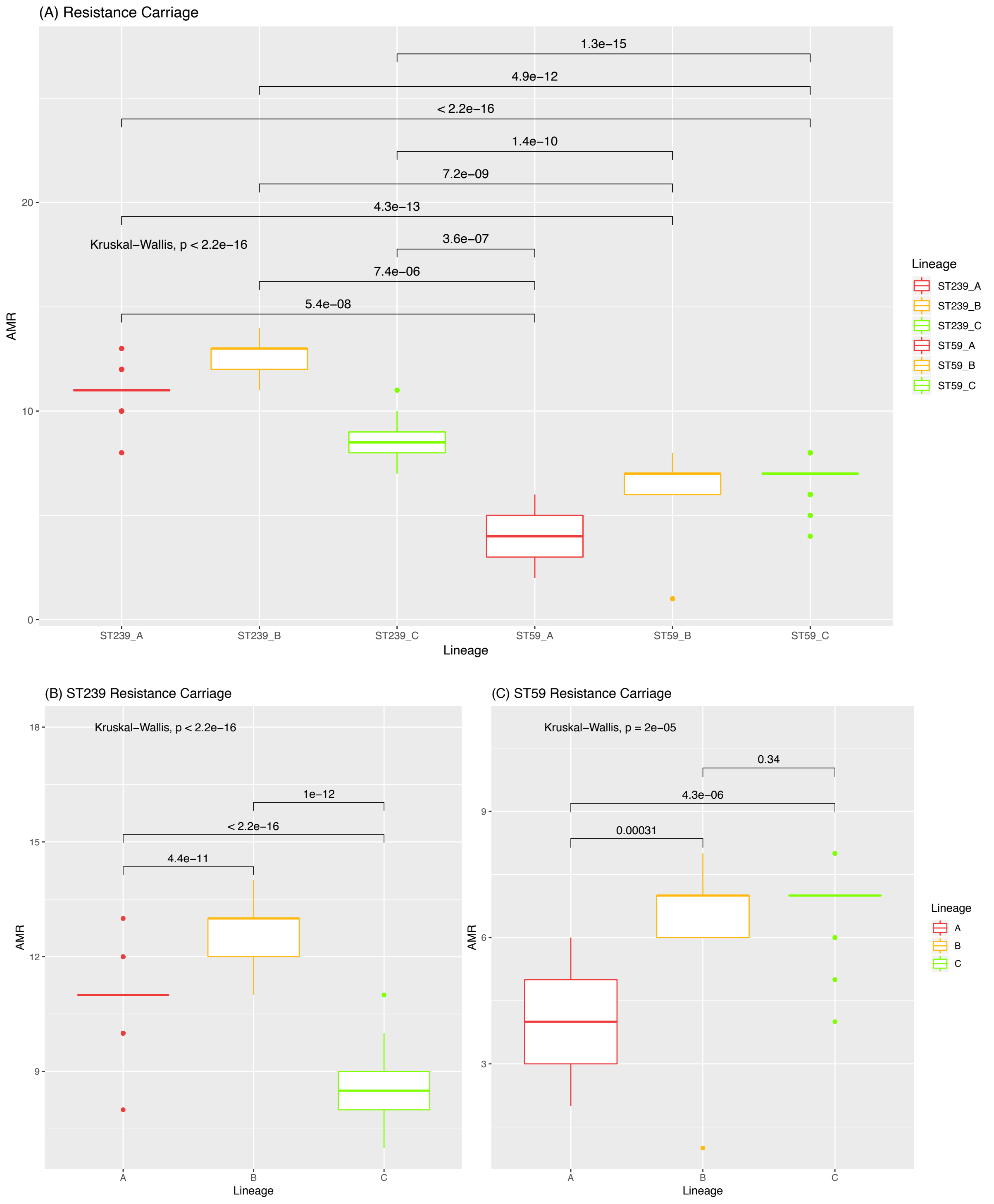

Fig S9: **Number of resistance (AMR) genes carried (A) between ST sub-lineages and within (B) ST239 and (C) ST59 sub-lineages.**

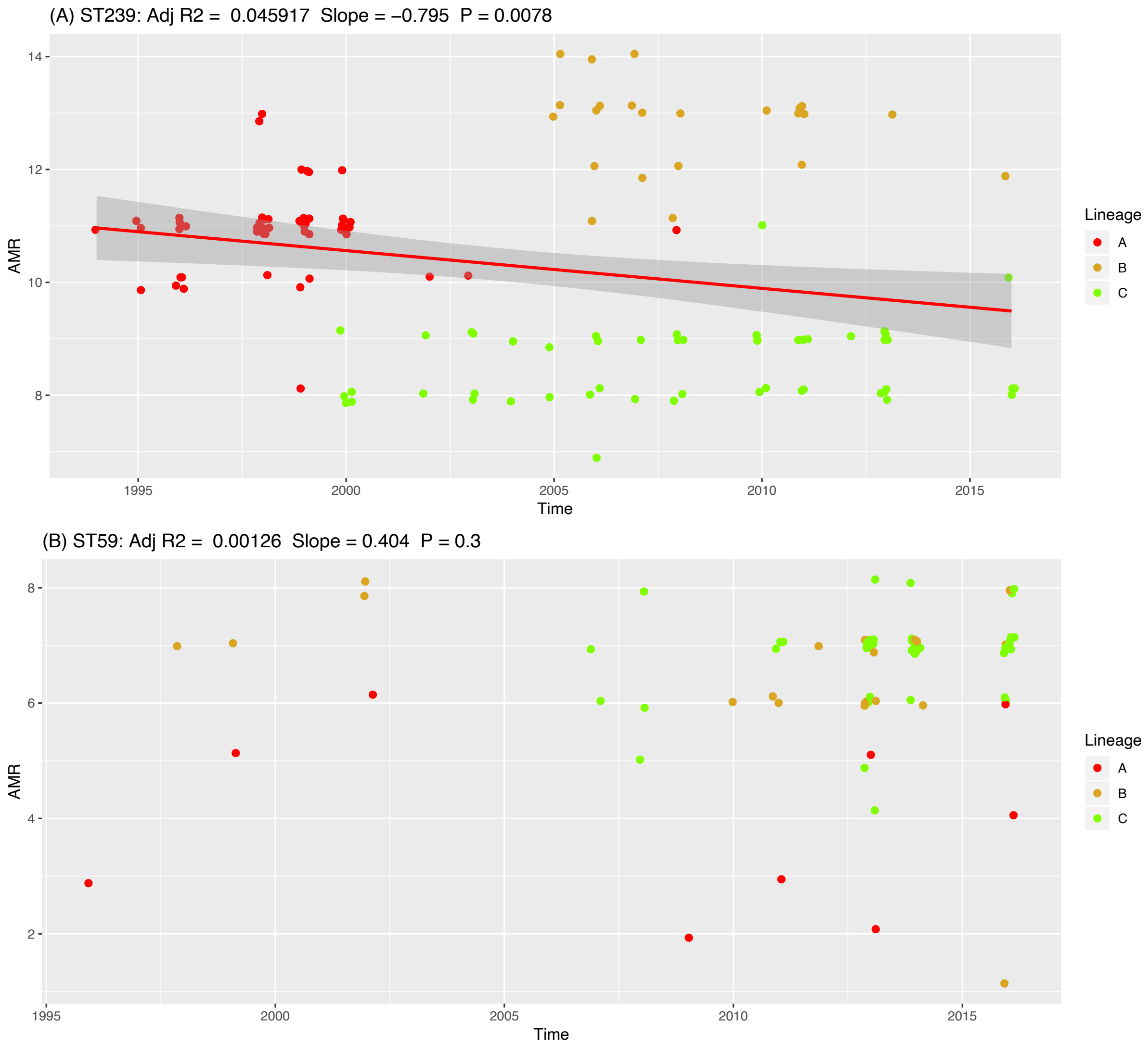

Fig S10: Changing trends of antimicrobial resistance (AMR) mutations/elements within (A) ST239 and (B) ST59 lineages over time. Number of genotypic resistance determinants per isolates from 1994 to 2016. Overlapping points are displayed with random jitter. Regression lines are provided for significant correlations.

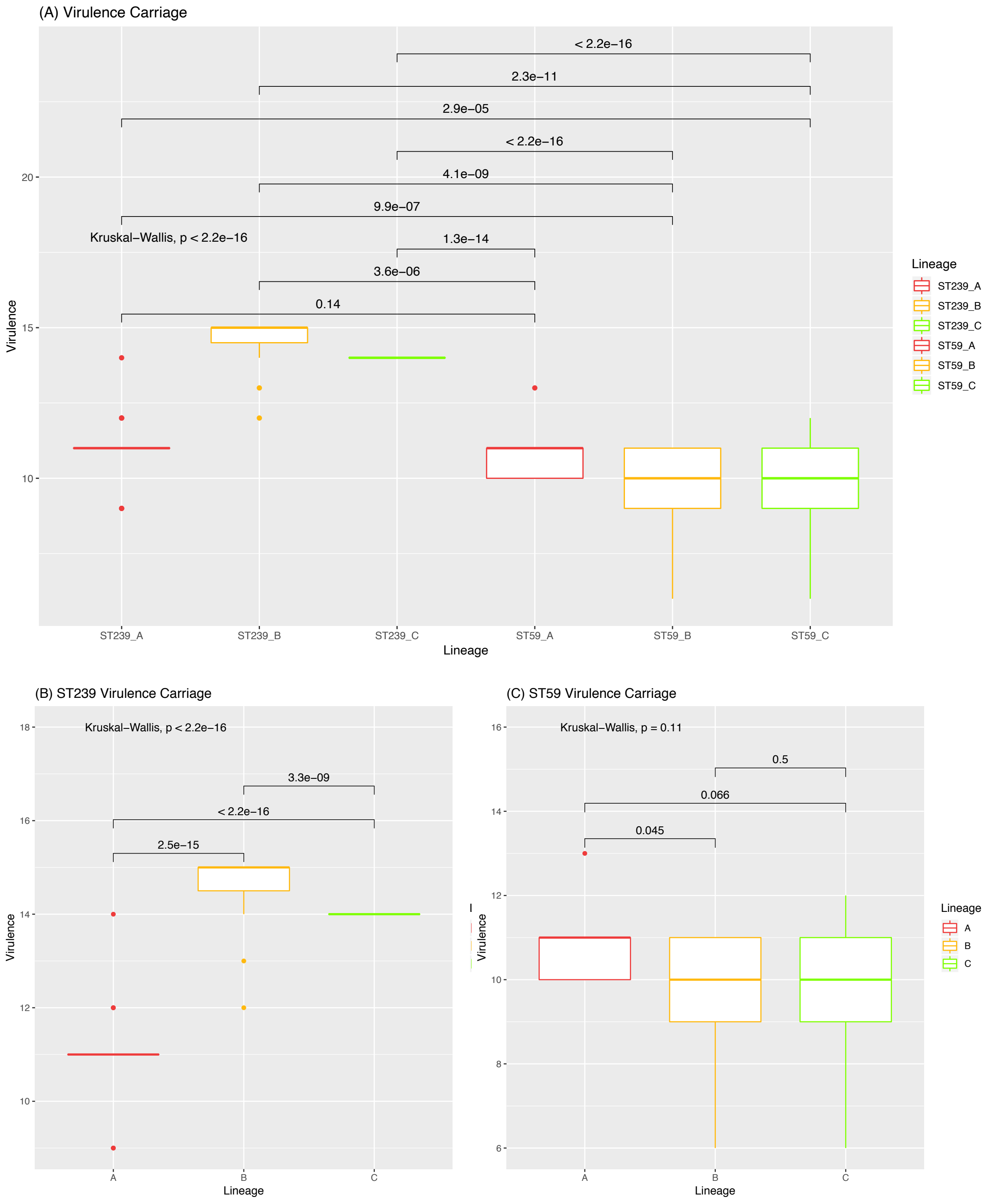

Fig S11: **Number of virulence genes carried (A) between ST sub-lineages and within (B) ST239 and (C) ST59 sub-lineages.**

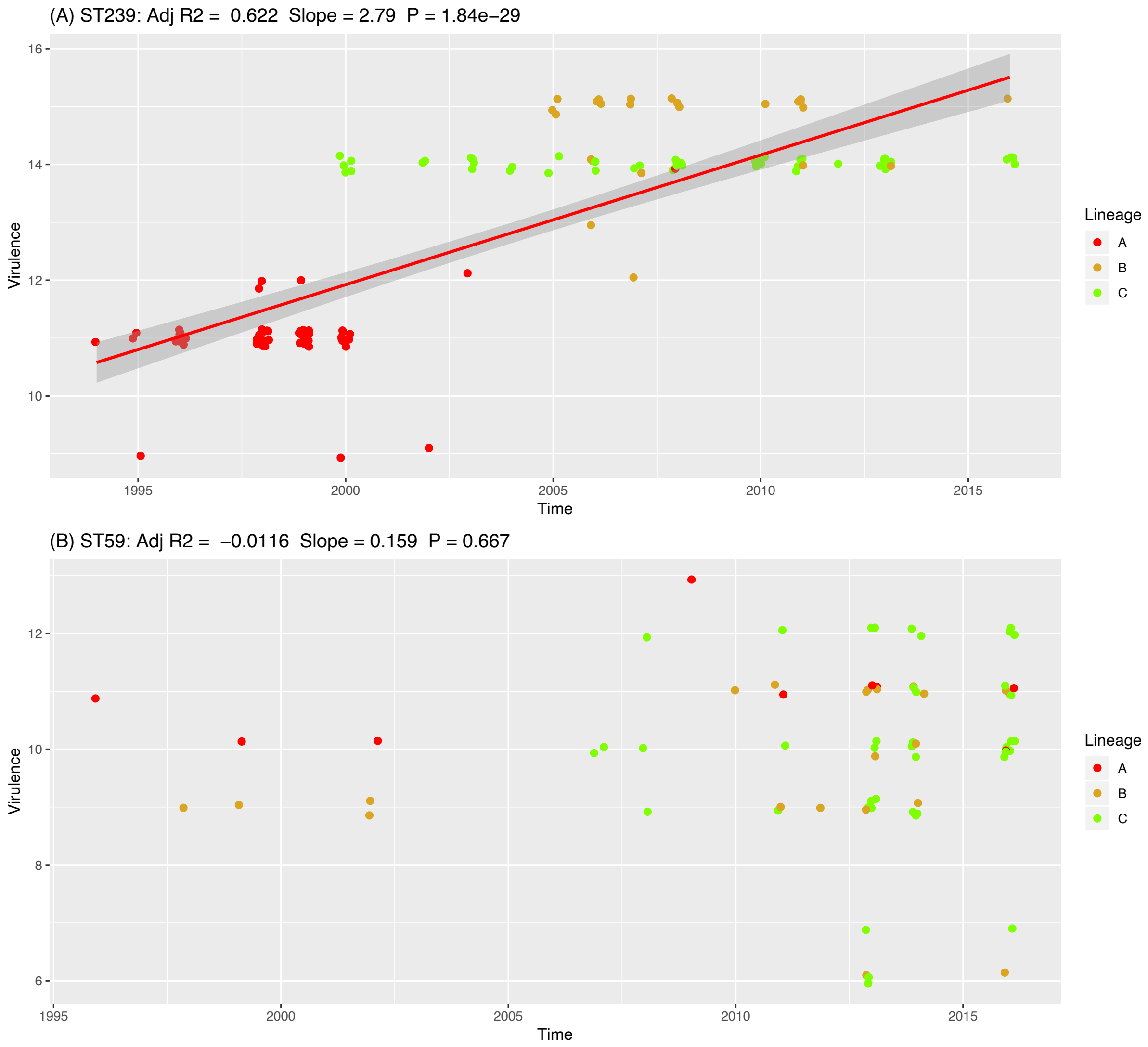

Fig S12: Trends of virulence mutations/elements within Chinese (A) ST239 and (B) ST59 lineages over time. Number of genotypic virulence determinants per isolates from 1994 to 2016. Overlapping points are displayed with random jitter. Regression lines are provided for significant correlations.

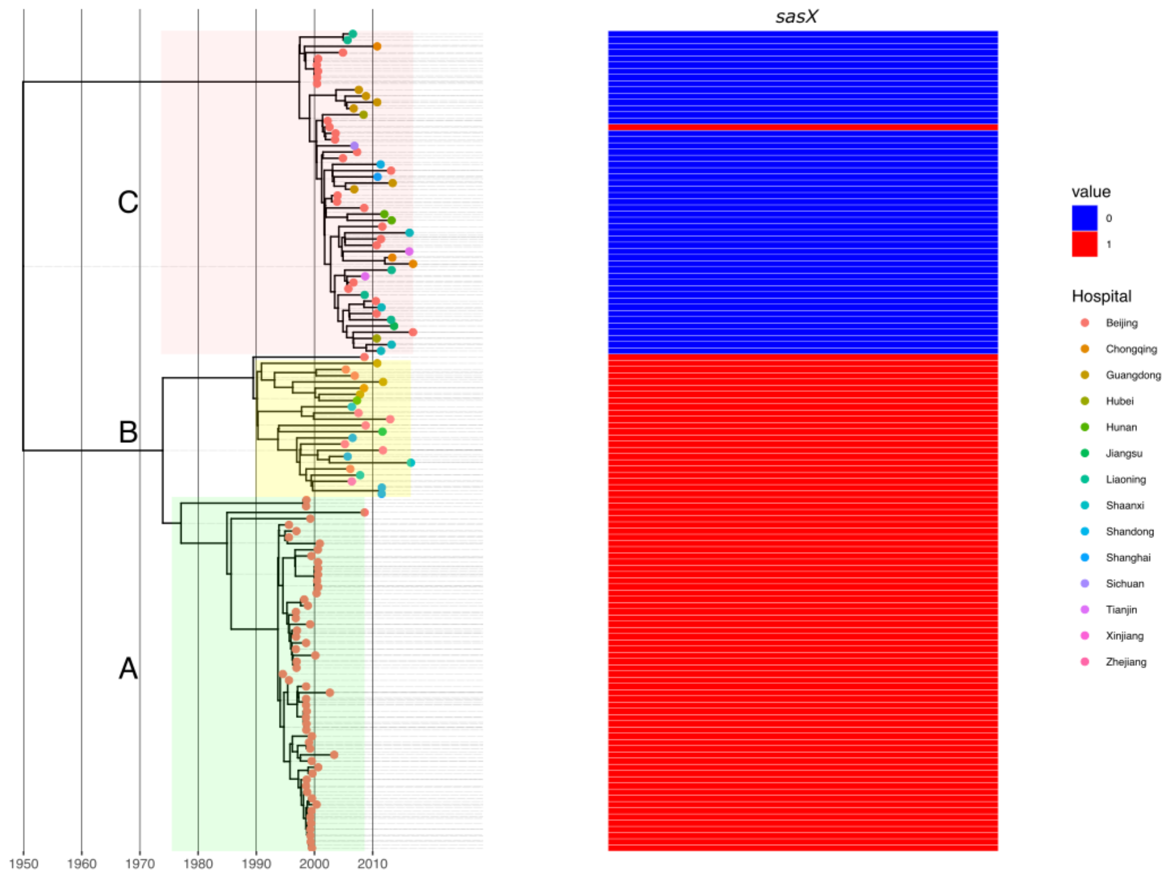

Fig S13: Core genome phylogeny for ST239 annotated for *sasX*. The coloured panel at right providing the concordance with the *sasX* gene distribution, as per the coloured legend.

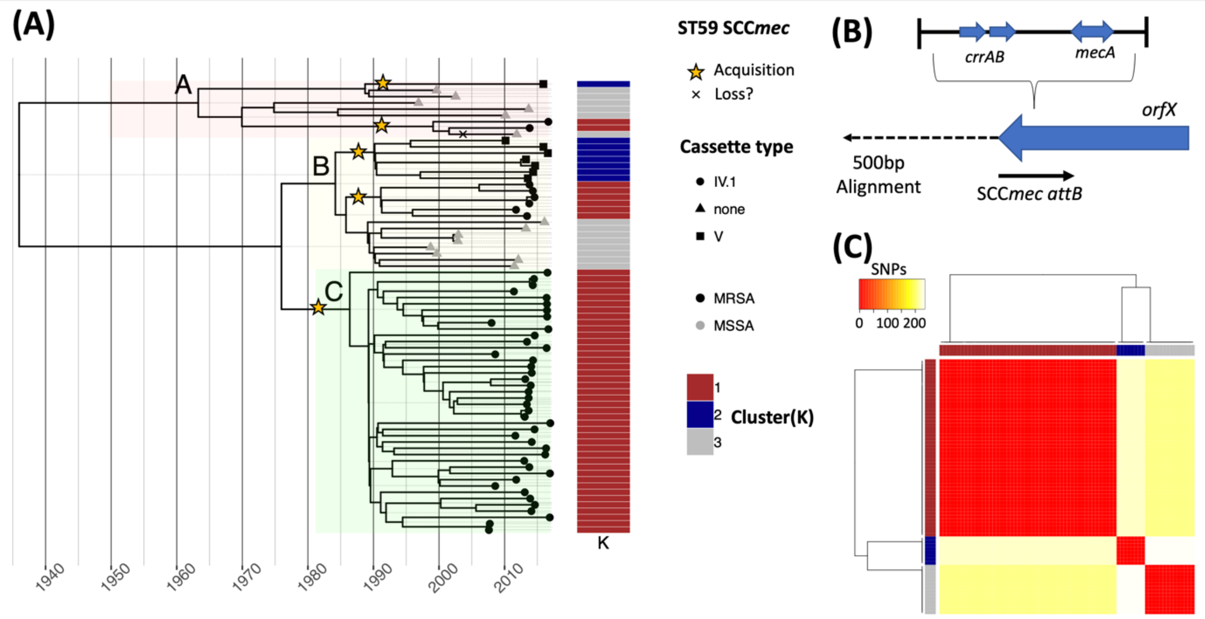

Fig S14: Methicillin resistance acquisition analyses. (A) Maximum clade credibility timed tree of ST59 isolates annotated by cassette cluster (panel). (B) A region 500bp upstream of and including the SCC*mec* attachment site was extracted and aligned across isolates. (C) Hierarchical clustering was applied to the resulting alignment. The clustering indicates at least five independent acquisitions of the SCC*mec* across our 58 MRSA ST59 isolates within the last 40 years, indicated by stars. One potential loss is indicated by a cross ‘x’.

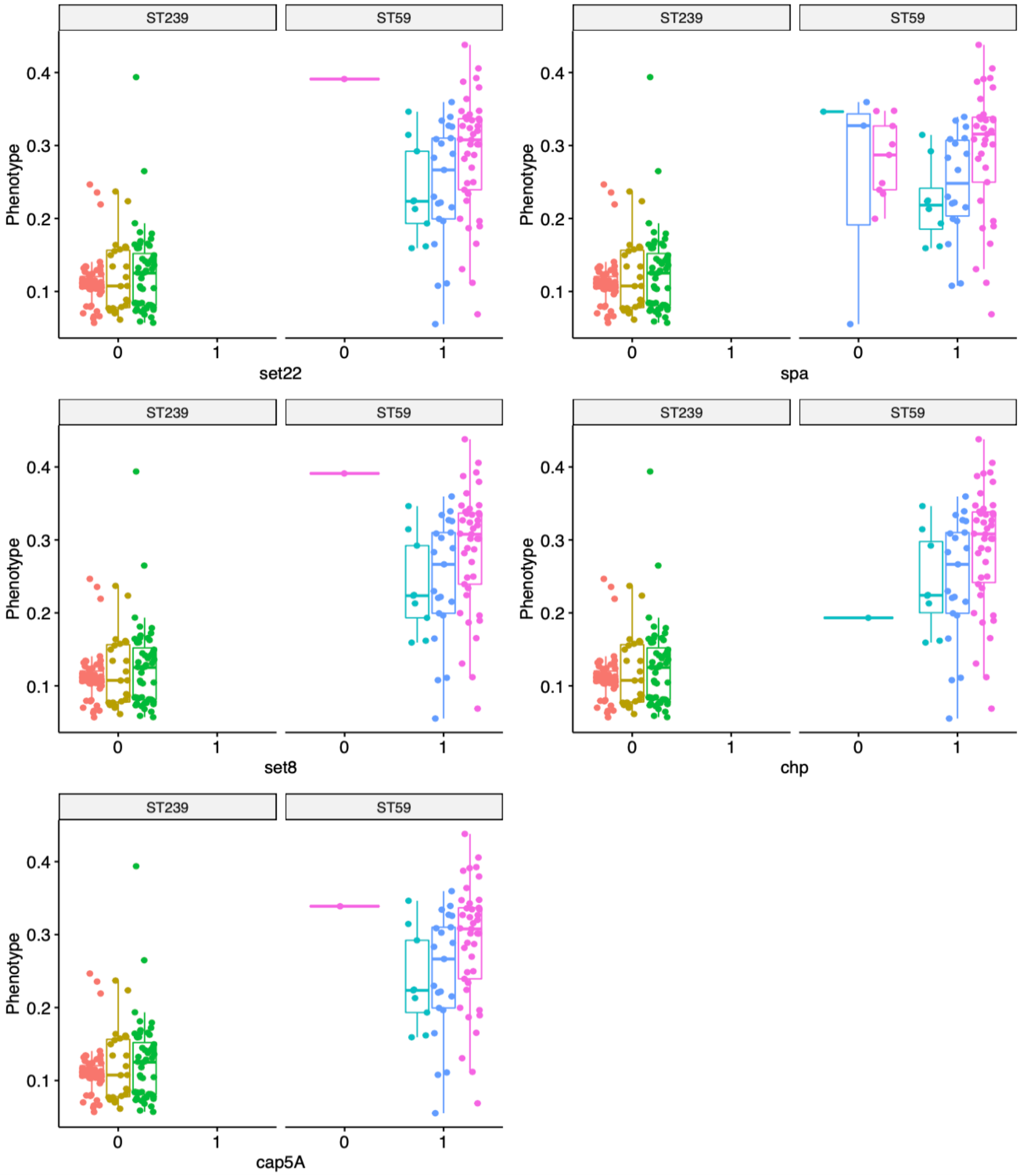

Fig S15: **Presence absence (1/0) of virulence genes (x-axis) against cell lysis phenotype.** Results are given for each of ST239 and ST59 for those genes which scored statistically highest following application of a generalized linear model (**Table S4**) for virulence genes present in ST59. Colours denote sub-lineages as follows: ST239-A (orange), ST239-B (mustard), ST239-C (green), ST59-A (turquoise), ST59-B (blue), ST59-C (pink).

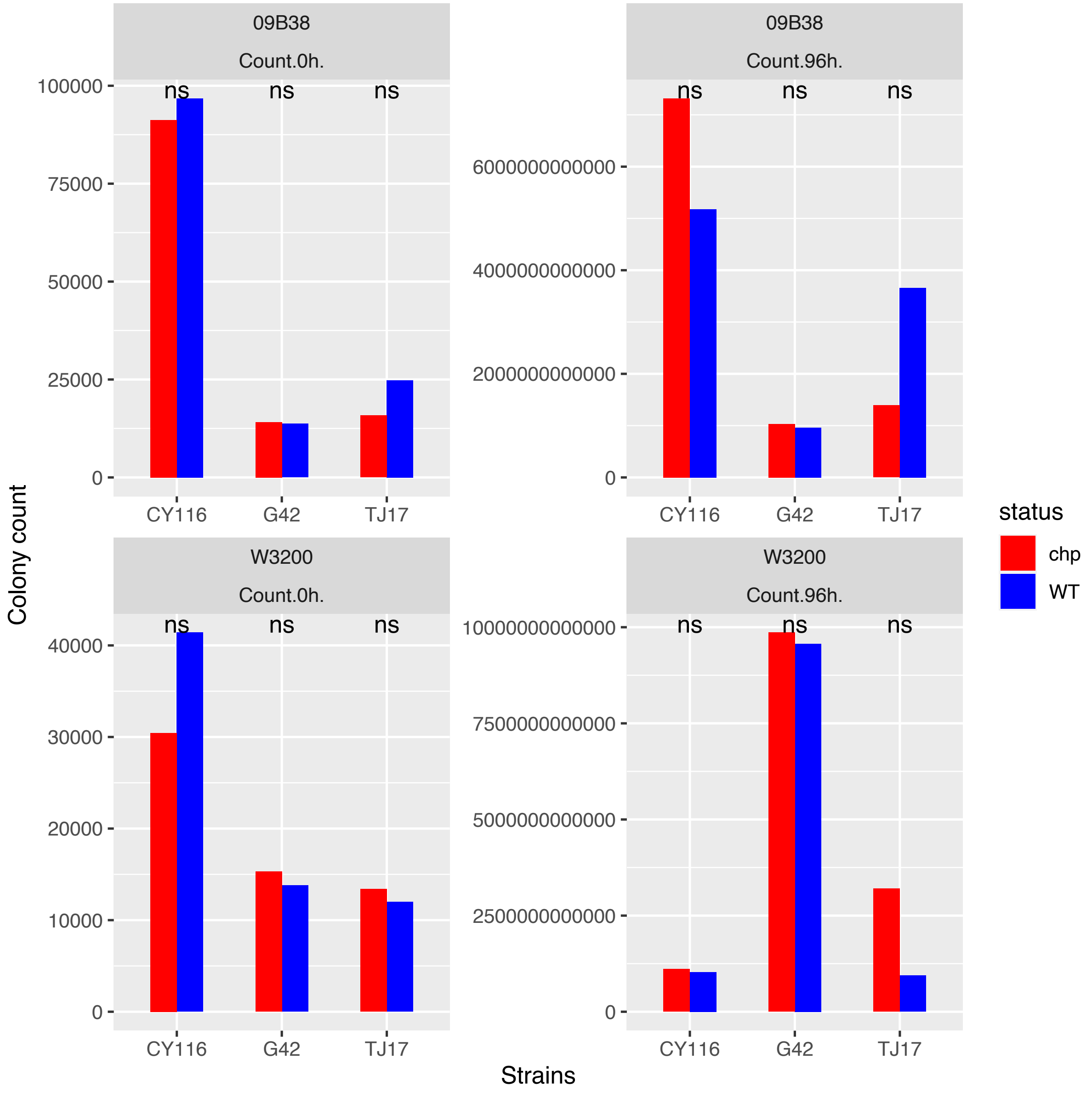

Fig S16: Colony counts at 0 hours (left) and 96 hours (right) for three ST59 isolates (wild-type and knock-out; blue and red respectively) compared to two possible ST239 control strains (top-09B38 and bottom-W3200). No timepoints/combinations are significantly different following Wilcoxon test.

Supplementary Tables

Table S1: [external file] Full metadata for 132 ST239 and 72 ST59 Chinese *S. aureus* isolates included in the phylogenetic analysis, including biofilm and virulence assay results with associated replicates. MLST, multilocus sequence type.

Table S2: Inferred tMRCA, clock rates and marginal likelihoods inferred by Bayesian dating analyses applied to the recombinant free core genome alignment across ST239 and ST59 isolates.

**ST239**	**Population Model**					
	**Coalescent Bayesian Skyline**			**Exponential Population Growth**		
Clock model	Strict	Relaxed Lognormal	Relaxed Exponential	Strict	Relaxed Lognormal	Relaxed Exponential
tMRCA	1948.9 (1942.4-1955.4)	1958.7 (1932.2-1985.2)	1959.4 (1932.6-1986.1)	1951.8 (1945.5-1958.0)	1988.1 (1982.6-1993.5)	1985.5 (1981.2-1989.8)
Clock rate	$1.8x10^{-6}$ ($1.7x10^{-6}$ - $2.0x10^{-6}$)	$2.6x10^{-6}$ ($2.1x10^{-6}$ - $3.1x10^{-6}$)	$2.7x10^{-6}$ ($2.2x10^{-6}$ - $3.2x10^{-6}$)	$1.9x10^{-6}$ ($1.7x10^{-6}$ - $2.0x10^{-6}$)	$3.0x10^{-6}$ ($2.2x10^{-6}$ - $4.3x10^{-6}$)	$2.8x10^{-6}$ ($2.2x10^{-6}$ - $3.4x10^{-6}$)
Marginal Likelihood	-2808993.957	-2809116.472	-2809169.438	-2809011.379	-2809115.283	-2809053.000
**ST59**	**Population Model**					
	**Coalescent Bayesian Skyline**			**Exponential Population Growth**		
Clock model	Strict	Relaxed Lognormal	Relaxed Exponential	Strict	Relaxed Lognormal	Relaxed Exponential
tMRCA	1933.8 (1921.0-1946.6)	1931.5 (1913.5-1949.4)	1932.0 (1887.4-1976.6)	1947.5 (1938.1-1956.8)	1955.1 (1942.9-1967.2)	1975.4 (1964.6-1986.2)
Clock rate	$1.2x10^{-6}$ ($1.0x10^{-6}$ - $1.4x10^{-6}$)	$1.2x10^{-6}$ ($0.9x10^{-6}$ - $1.4x10^{-6}$)	$1.7x10^{-6}$ ($1.0x10^{-6}$ - $2.5x10^{-6}$)	$1.4x10^{-6}$ ($1.2x10^{-6}$ - $1.5x10^{-6}$)	$1.4x10^{-6}$ ($1.2x10^{-6}$ - $1.7x10^{-6}$)	$2.0x10^{-6}$ ($1.3x10^{-6}$ - $2.8x10^{-6}$)
Marginal Likelihood	-2770579.818	-2770660.236	-2770796.468	-2770703.369	-2770694.160	-2770805.845

Table S3: The distribution of mobile genetic elements (MGEs) in different sub-lineages. The percentage represents the incidence of each MGE.

ST	MGEs
ST239	SCCmec III (100%), Tn5801_like (100%), IS256 (100%), vSaA (100%), vSaB (100%), IS1181 (99.2%), Tn552 (97.7%), phiPT1028_like (90.2%), IS431 (85.6%), phiSPbeta_like (71.2%), SaPI1 (55.3%), phiPVL_CN125_like (43.9%), Tn554_like (37.9%), SCCmercury (20.5%), phi53_like (27.3%), P1_tetk (18.9%), phiSA13_like (17.4%), phiNM3_like (17.4%), phiR1988_like (12.9%), phiN315_like (6.8%), phiJB_like (3.8%), P3 (3.0%), phinm2 (2.3%), P7_tw20_2 (2.3%), phiStauST398_4_like (1.5%), phi77_like (1.5%), phi187_like (0.8%), phiPV83_like (0.8%), phiIPla35_like (0.8%), phi92_like (0.8%), phitp310_1_like (0.8%), phitp310_3_like (0.8%), phi5967PVL_like (0.8%), phi2958PVL_like (0.8%), phiP954_like (0.8%), phiSalmon_SJ46_like (0.8%)
ST59	vSaA (100%), vSaB (100%), IS1216V (91.7%), Tn552_like (90.3%), Tn551 (88.9%), MESpm1_like (84.7%), SaPI3 (79.2%), IS431 (79.2%), IS1272 (69.4%), SCCmec IV (69.4%), phiPT1028_like (56.9%), P1_tetk (55.6%), phiNM3_like (31.9%), phi5967PVL_like (16.7%), phiPVL_like (12.5%), P4 (12.5%), SCCmec V (11.1%), phiJB_like (4.2%), IS256 (4.2%), phi88_like (2.8%), phiN315_like (2.8%), phi2958PVL_like (1.4%), phitp310_1_like (1.4%), phinm2_like (1.4%), phiMR11_like (1.4%), phiJS01_like (1.4%), SaPI4 (1.4%), P2_ermC (1.4%), P3 (1.4%), P5 (1.4%), P6 (1.4%)

Table S4: Bonferroni corrected *p*-values following fitting of a generalized linear model (GLM) for VFDB virulence gene presence / absence against cell lysis phenotype.

VFDB Gene	Std.Error	Z-value	Pr(>|z|)	Bonf.Correct	No.ST239	No.ST59
set22	3.34421135	7.54134576	4.6515E-14	3.07E-12	0	71
set8	3.34421135	7.54134576	4.6515E-14	3.07E-12	0	71
cap5A	3.43227224	7.5225914	5.3701E-14	3.5443E-12	0	71
spaA	2.38413536	7.45840696	8.7575E-14	5.7799E-12	0	59
**chp**	**3.71585539**	**7.44794792**	**9.4803E-14**	**6.257E-12**	**0**	**71**
capA	3.76683365	-7.4180698	1.1884E-13	7.8434E-12	132	0
lukD	3.76683365	-7.4180698	1.1884E-13	7.8434E-12	132	0
lukE	3.76683365	-7.4180698	1.1884E-13	7.8434E-12	132	0
set36	3.76683365	-7.4180698	1.1884E-13	7.8434E-12	132	0
set37	3.76683365	-7.4180698	1.1884E-13	7.8434E-12	132	0
set39	3.76683365	-7.4180698	1.1884E-13	7.8434E-12	132	0
set40	3.76683365	-7.4180698	1.1884E-13	7.8434E-12	132	0
splA	3.76683365	-7.4180698	1.1884E-13	7.8434E-12	132	0
splB	3.76683365	-7.4180698	1.1884E-13	7.8434E-12	132	0
splC	3.76683365	-7.4180698	1.1884E-13	7.8434E-12	132	0
splD	3.76683365	-7.4180698	1.1884E-13	7.8434E-12	132	0
vWbp	3.76683365	-7.4180698	1.1884E-13	7.8434E-12	132	0
aur	3.76683365	7.41806979	1.1884E-13	7.8434E-12	0	72
set23	3.76683365	7.41806979	1.1884E-13	7.8434E-12	0	72
set24	3.76683365	7.41806979	1.1884E-13	7.8434E-12	0	72
set25	3.76683365	7.41806979	1.1884E-13	7.8434E-12	0	72
sbi	3.74390311	-7.4084227	1.2781E-13	8.4355E-12	131	0
sraP	2.38923653	7.39319655	1.4334E-13	9.4605E-12	0	54
seb	2.32829293	7.37491374	1.6445E-13	1.0854E-11	0	57
set33	3.86196159	-7.3410517	2.1192E-13	1.3987E-11	129	0
map	3.38400078	-7.3409638	2.1206E-13	1.3996E-11	124	0
set35	3.99039227	-7.3047907	2.777E-13	1.8328E-11	129	0
set32	4.06499368	-7.298973	2.8997E-13	1.9138E-11	130	0
sbnA	2.40440734	-7.1699488	7.5026E-13	4.9517E-11	132	25
sbnB	2.40440734	7.16994879	7.5026E-13	4.9517E-11	0	47
ebh	2.44332358	-6.4185171	1.3761E-10	9.0821E-09	102	0
coa	1.86710072	6.19188549	5.9449E-10	3.9236E-08	14	60
geh	2.53512256	5.98086592	2.2195E-09	1.4649E-07	42	72
lukF.PV	2.82431802	5.96921231	2.384E-09	1.5735E-07	0	28
lukS.PV	2.82431802	5.96921231	2.384E-09	1.5735E-07	0	28
fnbA	3.09484172	-5.508238	3.6244E-08	2.3921E-06	73	0
sdrC	2.81858937	5.07069178	3.9637E-07	2.6161E-05	0	21
scn	3.68458321	4.76858247	1.8553E-06	0.00012245	78	72
clfB	2.01575661	4.45564476	8.3641E-06	0.00055203	5	26
sea	1.89002811	-4.3270209	1.5114E-05	0.00099752	74	8

sirB	2.17884282	-3.4369411	0.00058832	0.03882936	132	49
sirC	2.17884282	3.43694107	0.00058832	0.03882936	0	23
fnbB	3.95233712	-3.2345083	0.00121852	0.08042258	29	0
selk	2.18295861	-2.8060885	0.00501469	0.33096965	125	57
selq	2.13931601	-2.6223486	0.00873261	0.57635201	124	57
adsA	7.55828882	0.04478418	0.96427934	1	131	71
clfA	2.74543978	-1.460538	0.14414228	1	20	3
eap.map	6.76736979	-1.2260924	0.22016389	1	132	70
ebp	13.8897	-1.2686722	0.20455799	1	132	71
hld	47.9090897	0.92946124	0.3526501	1	131	72
hly.hla	7.12462517	-0.1701287	0.86490891	1	130	72
icaC	8.41054926	0.30695847	0.75887498	1	130	72
isdD	23.6947433	0.63662517	0.52436902	1	131	72
isdH	3.4620454	-0.8937032	0.37148069	1	132	64
SACOL0507	9.33252373	-0.4894906	0.62449443	1	132	71
sak	1.60175011	1.87860665	0.06029822	1	78	49
sasC	10.7501615	-1.1308275	0.2581277	1	132	71
set30	6.59894828	-0.7879876	0.43070394	1	130	72
set31	5.84323315	-1.7538312	0.07945943	1	130	71
set34	5.84323315	-1.7538312	0.07945943	1	130	71
selp	14.2729822	-0.8306972	0.40614472	1	0	2
set19	3.88741347	1.19709518	0.23126946	1	0	6
icaB	8.41054926	-0.3069585	0.75887498	1	2	0
sdrD	9.32714575	0.73147765	0.46448745	1	1	0
splE	2.97117897	-1.9913491	0.04644252	1	24	0
splF	7.61516163	-2.015788	0.04382215	1	10	0

Table S5: Relative adaptive difference (S), fitness (F) and fitness cost (C) estimated from the *in vitro* competition experiment between ST59-WT and ST59 Δ*chp*.

Experimental Strains	Control Strain (ST239)	S	F	C
G42-Δ*chp* / G42 (ST59)	W3200	-0.004	99.6	0.4
CY116-Δ*chp* / CY116 (ST59)		0.01	101.4	-1.4
TJ17-Δ*chp* / TJ17 (ST59)		0.07	106.5	-6.5
G42-Δ*chp* / G42 (ST59)	09B38	0.003	100.3	-0.3
CY116-Δ*chp* / CY116 (ST59)		-0.03	97.0	3
TJ17-Δ*chp* / TJ17 (ST59)		0.02	102.40	-2.4
